# Beyond a platform protein for the degradosome assembly: The Apoptosis-Inducing Factor as an efficient nuclease involved in chromatinolysis

**DOI:** 10.1093/pnasnexus/pgac312

**Published:** 2022-12-26

**Authors:** Nerea Novo, Silvia Romero-Tamayo, Carlos Marcuello, Sergio Boneta, Irene Blasco-Machin, Adrián Velázquez-Campoy, Raquel Villanueva, Raquel Moreno-Loshuertos, Anabel Lostao, Milagros Medina, Patricia Ferreira

**Affiliations:** Departamento de Bioquímica y Biología Molecular y Celular, Facultad de Ciencias, Universidad de Zaragoza, Zaragoza 50009, Spain; Instituto de Biocomputación y Física de Sistemas Complejos, BIFI (GBsC-CSIC Joint Unit), Universidad de Zaragoza, Zaragoza 50018, Spain; Departamento de Bioquímica y Biología Molecular y Celular, Facultad de Ciencias, Universidad de Zaragoza, Zaragoza 50009, Spain; Instituto de Biocomputación y Física de Sistemas Complejos, BIFI (GBsC-CSIC Joint Unit), Universidad de Zaragoza, Zaragoza 50018, Spain; Instituto de Nanociencia y Materiales de Aragón (INMA), CSIC-Universidad de Zaragoza, Zaragoza 50009, Spain; Laboratorio de Microscopias Avanzadas (LMA), Universidad de Zaragoza, Zaragoza 50018, Spain; Departamento de Bioquímica y Biología Molecular y Celular, Facultad de Ciencias, Universidad de Zaragoza, Zaragoza 50009, Spain; Departamento de Bioquímica y Biología Molecular y Celular, Facultad de Ciencias, Universidad de Zaragoza, Zaragoza 50009, Spain; Departamento de Bioquímica y Biología Molecular y Celular, Facultad de Ciencias, Universidad de Zaragoza, Zaragoza 50009, Spain; Instituto de Biocomputación y Física de Sistemas Complejos, BIFI (GBsC-CSIC Joint Unit), Universidad de Zaragoza, Zaragoza 50018, Spain; Aragón Institute for Health Research (IIS Aragón), Zaragoza, Zaragoza 50009, Spain; Biomedical Research Networking Centre for Liver and Digestive Diseases (CIBERehd), Madrid 28029, Spain; Departamento de Bioquímica y Biología Molecular y Celular, Facultad de Ciencias, Universidad de Zaragoza, Zaragoza 50009, Spain; Instituto de Biocomputación y Física de Sistemas Complejos, BIFI (GBsC-CSIC Joint Unit), Universidad de Zaragoza, Zaragoza 50018, Spain; Departamento de Bioquímica y Biología Molecular y Celular, Facultad de Ciencias, Universidad de Zaragoza, Zaragoza 50009, Spain; Instituto de Biocomputación y Física de Sistemas Complejos, BIFI (GBsC-CSIC Joint Unit), Universidad de Zaragoza, Zaragoza 50018, Spain; Instituto de Nanociencia y Materiales de Aragón (INMA), CSIC-Universidad de Zaragoza, Zaragoza 50009, Spain; Laboratorio de Microscopias Avanzadas (LMA), Universidad de Zaragoza, Zaragoza 50018, Spain; Fundación ARAID, Aragón, Zaragoza 50018, Spain; Departamento de Bioquímica y Biología Molecular y Celular, Facultad de Ciencias, Universidad de Zaragoza, Zaragoza 50009, Spain; Instituto de Biocomputación y Física de Sistemas Complejos, BIFI (GBsC-CSIC Joint Unit), Universidad de Zaragoza, Zaragoza 50018, Spain; Departamento de Bioquímica y Biología Molecular y Celular, Facultad de Ciencias, Universidad de Zaragoza, Zaragoza 50009, Spain; Instituto de Biocomputación y Física de Sistemas Complejos, BIFI (GBsC-CSIC Joint Unit), Universidad de Zaragoza, Zaragoza 50018, Spain

**Keywords:** human Apoptosis-Inducing Factor, DNA–degradosome complex, nuclease activity, chromatinolysis

## Abstract

The Apoptosis-Inducing Factor (AIF) is a moonlighting flavoenzyme involved in the assembly of mitochondrial respiratory complexes in healthy cells, but also able to trigger DNA cleavage and parthanatos. Upon apoptotic-stimuli, AIF redistributes from the mitochondria to the nucleus, where upon association with other proteins such as endonuclease CypA and histone H2AX, it is proposed to organize a DNA–degradosome complex. In this work, we provide evidence for the molecular assembly of this complex as well as for the cooperative effects among its protein components to degrade genomic DNA into large fragments. We have also uncovered that AIF has nuclease activity that is stimulated in the presence of either Mg^2+^ or Ca^2+^. Such activity allows AIF by itself and in cooperation with CypA to efficiently degrade genomic DNA. Finally, we have identified TopIB and DEK motifs in AIF as responsible for its nuclease activity. These new findings point, for the first time, to AIF as a nuclease able to digest nuclear dsDNA in dying cells, improving our understanding of its role in promoting apoptosis and opening paths for the development of new therapeutic strategies.

Significance Statement The cell state determines whether the Apoptosis-Inducing Factor (AIF) will act as a prolife or a prodeath effector. Under some pathological conditions, its prolife NADH oxidase activity can contribute to the proliferation and aggressiveness of certain cancers. Moreover, mutations in its gene can also compromise its prolife functions by producing phenotypes associated with mitochondrial encephalomyopathies, as well as boosting its cell-death activity. As a prodeath effector, AIF is proposed to be the platform for the assembly of a multiprotein complex that enhances DNA degradation. We provide insights into the molecular formation of such complex and how it improves chromatinolysis. Moreover, we show that AIF is inherently able to degrade DNA. Knowing more about these features is essential to better understand the AIF apoptotic function and to treat pathological phenotypes.

## Introduction

The Apoptosis-Inducing Factor (AIF) is a mitochondrial flavoprotein that contributes to both cellular life and death (1). Under physiological conditions, AIF is anchored to the mitochondrial inner membrane, facing the intermembrane space, where it interacts with CHCHD4 (coiled-coil-helix-coiled-coil-helix domain containing 4)—a key assembly factor for multisubunit respiratory electron transport chain complexes—and plays an essential role in the maintenance of mitochondrial structure and oxidative phosphorylation (2, 3). Conversely, under pathological conditions, AIF is one of the main effectors of caspase-independent necroptosis. This manner of programmed cell death (PCD) involves the redistribution of AIF to the nuclear compartment where it induces chromatin condensation, large-scale DNA fragmentation (≈20 to 50 kb) and DNA loss (4, 5).

In response to a variety of cytotoxic stimuli, oxidative stress or DNA alkylating agents, hyperactivation of the nuclear polymerase-1-dependent cell death (PARP-1) initiates a signaling cascade that provokes mitochondrial damage. This results in the release of the soluble truncated proapoptotic AIF form—AIF_Δ101_ in humans, cleaved by activated calpains proteases—from the mitochondria to the cytosol. Interestingly, the subsequent translocation of AIF to the nucleus is hindered or promoted through its physical interaction with either the heat shock protein-70 (HSP70) or cyclophilin A (CypA) respectively, by mechanisms still not completely understood (6, 7). Once in the nucleus, the lethal activity of AIF is said to rely on its ability to associate or activate nucleases, since AIF apparently lacks genuine endonuclease activity, despite being able to bind chromatin (1, 8–10). Previous results showed that the synchronized action of AIF, phosphorylated histone H2AX and nuclease CypA is required to provoke chromatin remodeling and DNA loss under alkylating DNA damage upon MNNG (methylnitronitrosoguanidine)-mediated necroptosis (4). Indeed, there is strong evidence of nuclear AIF/H2AX interplay (4). This might, in some way, promote the activation of the latent nuclease activity of CypA, as well as support the association of AIF, H2AX, and CypA in a multiprotein complex commonly referred to as the degradosome (4). It is worth nothing that H2AX participates in DNA damage repair in response to DNA double-strand breaks, while simultaneously playing a key role in PCD by likely inducing DNA restructuration to improve accessibility to endonucleases (11, 12). AIF might also cooperate directly with CypA to promote neuronal death in response to different cellular stress conditions (13, 7). In particular, upon cerebral hypoxia–ischemia, the cytosolic AIF/CypA interaction is proposed to favor the nuclear cotranslocation of both proteins (7). Nonetheless, further studies are needed to clarify the molecular mechanisms through which these DNA-degrading complexes execute their lethal action as a function of the particular apoptotic stimuli and even the cellular and tissue nature.

In this scenario, AIF has been proposed as a platform for the assembly of an active DNA–degradosome complex during PCD (14). This is supported by the fact that AIF may concurrently interact with all of the degradosome components through different surface regions. Thus, AIF would interact with H2AX through a proline-rich motif (aa 544 to 554 in *Homo sapiens* AIF, part of the flexible regulatory C-loop) in its apoptotic C-terminal domain that is essential for its apoptogenic capacity (4). On its part, CypA would specifically associate to the 370 to 394 amino acid region within the NADH-binding domain of *H. sapiens* AIF (15). Finally, AIF interacts with DNA in a sequence-independent manner based on electrostatic interactions around a positively charged protein crown (10, 16). However, up to date no in vitro or in vivo evidence exists for the formation of this nuclear chromatinolytic degradosome complex, despite a plausible theoretical molecular model (14). Furthermore, little is known about the physiological relevance of the mutual interactions among these proteins, their cooperativity or how they operate to promote chromatinolysis.

Using a combination of biophysical and molecular biology methodologies, we here provide insights into the formation of DNA–degradosome assemblies at the molecular level, being able to enlarge their efficiency on DNA chromatinolysis. In addition, we also demonstrate AIF’s capability to enact DNA nuclease activity by itself, unregimented by either the endonuclease CypA or the histone H2AX.

## Materials and Methods

### Production of recombinant proteins

AIF_∆101_ (Apoptosis-Inducing Factor, UniProtKB O95831) and its Y443A, K446A, R449A, R450A, R451A, H454S, K510A/K518A, D489A/K518A, and K518A/E522A variants, CypA (PPIA, peptidyl prolyl isomerase A, UniProtKB P62937) and Histone H2AX (UniProtKB P16104) from *H. sapiens* were heterologously expressed in *Escherichia coli* C41 (DE3) as recombinant proteins with an N-terminal His_6_-tag using the pET-28a(+) vector. Proteins were expressed and purified as described in the supplementary materials.

### Clear native (CN) and 2D denaturing electrophoresis

Mixtures of AIF_∆101_ with its different nuclear partners were incubated for 15 min in 50 mM potassium phosphate, pH 7.4, at 25°C. Samples were then separated by CN-PAGE gradient electrophoresis (polyacrylamide concentration gradient 4% to 20%). Afterwards, gels were processed for 2D SDS-PAGE and western blotting (WB) detection. See Supplementary Material for more details.

### Size-exclusion chromatography

AIF_Δ101_ was incubated with its different nuclear protein partners (1:3 ratio) for 15 min at 25°C in 50 mM potassium phosphate, pH 7.4. Samples were then loaded into a Sephadex S-200 High Resolution (GE Healthcare) column connected to an Äkta Purifier HPLC system (GE Healthcare). Protein elution was performed in 50 mM potassium phosphate, 10 mM NaCl, pH 7.4, at a flow rate of 0.4 mL min^−1^. The column was previously calibrated with the GE Healthcare low molecular weight calibration kit (six proteins in the 6.4 to 160 kDa range). The obtained chromatograms were fitted to a set of Gaussian functions.

### AFM imaging

AFM measurements were carried out with a Cervantes FullMode Scanning Probe Microscope (Nanotec Electrónica S.L.) at room conditions. Mixtures of dsDNA–degradosome components were incubated on fresh cleaved mica pieces (Electron Microscopy Sciences) for 10 min at room temperature to achieve immobilization. AFM images were acquired using the intermittent force Jumping Mode (17) at low applied forces to minimize lateral forces and dragging effects (18). Silicon nitride AFM soft microlevers with ultrasharp 2 nm nominal final end tip radius exhibiting 0.01 to 0.03 N m^−1^ calibrated spring constants were used (MSNL; Bruker Probes). See Supplementary Material for more details.

### ITC measurements

The interaction among the dsDNA–degradosome components was assayed by ITC using an Auto-iTC200 calorimeter (MicroCal, Malvern-Panalytical). Typically, a 10-μM sample of the desired protein in the calorimetric cell was titrated with 100 μM of either dsDNA or any of the protein partners by performing 19 injections of 2 μL volume, spaced every 150 s, and mixed using a stirring speed of 750 rpm. The association constant (*K_a_*), the enthalpy change (∆H), and the binding stoichiometry (N) were estimated through non-linear least-squares regression data analysis of the experimental data employing a single ligand binding site model (1:1 protein:ligand stoichiometry) implemented in Origin 7.0 (19, 20). In general, errors in the determined parameters were ± 0.4 kcal mol^−1^ for ΔH and TΔS, ±0.1 kcal mol^−1^ for ΔG, and ± 30% for binding constants, *K*_a_ or *K*_d_. The assays for evaluating the buffer independent binding enthalpy and for determining the binding cooperativity to AIF_Δ101_ among ligands are described in the Supplementary Material. The assays with binary and ternary complexes for assessing heterotropic cooperativity were performed applying a similar protocol, but placing a mixture of AIF in complex with other partners at a certain molar ratio.

### Electrophoretic–mobility–shift assays

DNA retardation assays were carried out by mixing 500 ng of GeneRuler 100 bp dsDNA ladder (Thermo Scientific) with 6 µg of AIF, which were then incubated for 30 min at 25°C in 50 mM of potassium phosphate, pH 7.4. Samples were subsequently mixed with 2 µL of 6x DNA loading dye (Thermo Scientific), resolved by electrophoresis in 2% agarose gels and visualized by ethidium bromide staining (EtBr). Results were processed with a ChemiDoc™ XRS + System from BioRad (Quantity One).

### Nuclease activity assays

Assays were performed by mixing 250 ng of a double–stranded supercoiled pET-28a(+) plasmid or 500 ng of genomic DNA as substrate with 250 ng of purified AIF_Δ101_ (or 1 IU of DNase) in 20 mM Tris, pH 8.0 in a final volume of 10 µL, with 0.1 mM CaCl_2_ and 1 mM MgCl_2_ unless otherwise stated. Samples were incubated for 1 min at 37°C, unless otherwise stated, after which they were mixed with 2 µL of 6x DNA loading dye (Thermo Scientific) and subsequently incubated for 10 min at 65°C. Samples were then loaded onto a 1% or 0.7% agarose gel with EtBr and run for 1 or 4 hours at 90 or 60 V depending on whether they contained plasmid or genomic DNA. For the 4-hour long runs, gel chambers were immersed in an ice-bath to maintain a stable temperature. Human or mouse genomic DNA was extracted from HeLa or MEF cells, respectively, by means of a QIAamp DNA kit (Qiagen). To determine the influence on nuclease activity of the AIF_Δ101_ interaction with physiological partners, protein mixtures were preincubated for 15 min at 25°C prior to mixing with dsDNA. Genomic DNA integrity assays were performed on the 2200 TapeStation microfluidic platform (Genomic ScreenTape device, Agilent Technologies) by using a genomic ladder and following the manufacturer’s instructions. Integrities were quantified using DINs in a scale of 1 to 10 (from very degraded DNA to highly intact DNA). Additionally, densitometry was used to quantitate the percentage of degraded dsDNA and calculate the nuclease activity (see Supplementary Material for more details).

### Building of structural models of the DNA–degradosome multicomplex

Energetically optimized models for the degradosome assembly were built by subsequent steps of protein–protein docking and molecular dynamics simulations using experimental data to restrain binary interaction surfaces. Haddock and Graphite-Life Explorer servers were used to build the DNA–degradosome model (21, 22). Further details can be found in the Supplementary Material.

## Results and Discussion

### Visualizing the degradosome assembly at the molecular level

**Fig. 1.**
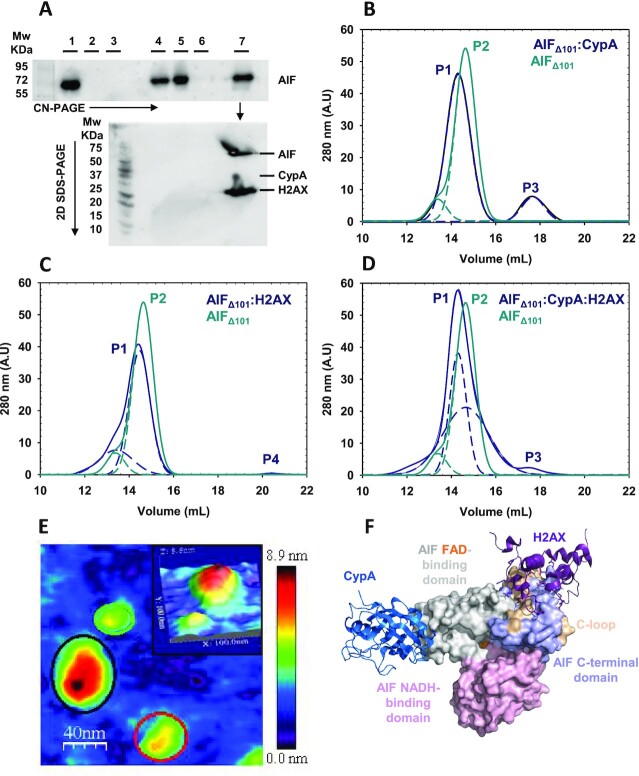
The degradosome quaternary organization. (A) Western blot analyses of degradosome composition. The top panel shows the high-resolution CN-PAGE separation of: Lane 1, AIF_Δ101_; lane 2, CypA; lane 3, H2AX; lane 4, hAIF_Δ101_:CypA (1:3 ratio); lane 5, AIF_Δ101_:H2AX (1:3 ratio); lane 6, CypA:H2AX (1:3 ratio); and lane 7, AIF_Δ101_:CypA:H2AX (1:3:3 ratio). Mixtures were incubated 15 min at 25°C in 50 mM potassium phosphate, pH 7.4, prior to electrophoresis separation. Then, the blot was probed with AIF_Δ101_-specific antibodies. A duplicated of the lane 7 was excised from the CN-PAGE and loaded into a second SDS-PAGE dimension (lower panel) to separate proteins by MW. The resulting protein spots were western-analyzed using anti-His-tag. Chromatographic elution profiles of mixtures of AIF_Δ101_ with (B) CypA, (C) H2AX, and (D) both proteins are shown in blue lines. In each mixture, a control profile for AIF_Δ101_ alone is shown in light green line. Samples were incubated at 1:3 or 1:3:3 ratios in binary and ternary mixtures, respectively, for 15 min at 25°C in 50 mM potassium phosphate, pH 7.4, before passing through a -Superdex 200 column using the same buffer supplemented with 10 mM NaCl. The respective different populations assigned by Gaussian analysis are depicted in dashed lines. (E) Representative AFM topography of an AIF_Δ101_:CypA:H2AX assembly. AFM image of a sample of AIF_Δ101_ incubated with CypA and H2AX (1:1:1 ratio) for 10 min. Red and green circles indicate AIF_Δ101_ and H2AX monomers, respectively, whereas the black circle stands for an AIF_Δ101_:CypA:H2AX assembly. Scan size 200 nm × 200 nm. The inset panel depicts zoom of a representative 3D AFM degradosome image at a scan size of 100 nm × 100 nm. In Figure S2, the different assemblies corresponding to monomers, dimers, and heterotrimers can be observed in larger areas of the sample. (F) Energetically optimized model for an AIF_Δ101_:CypA:H2AX assembly. Model based on experimental data to identify hot spot interaction surfaces and constructed by subsequent steps of protein–protein docking and molecular dynamics. CypA and H2AX are shown as cartoon colored in blue metallic and dark raspberry, respectively. AIF surfaces for FAD-binding, NADH-binding, and C-terminal domains are colored in gray, light pink, and light violet, respectively. The C-loop is highlighted in wheat and the FAD cofactor is shown as orange spheres. Starting PDB codes were 3K0M, 6K1K chain C, and 4BV6 for CypA, H2AX, and AIF, respectively.

In sight of accumulated evidences suggesting that AIF associates in the nucleus with CypA and H2AX to form a degradosome multicomplex, we used different methods to prove the ability of apoptotic AIF_Δ101_ to simultaneously assemble with CypA and H2AX in vitro. When incubated with 3-fold excess of either H2AX or CypA (16 and 18 kDa, respectively), or with both simultaneously, AIF_Δ101_ (58 kDa) migrated as a unique band that shifted toward higher apparent molecular weights (^app^MWs) in high-resolution clear native electrophoresis (CN-PAGE) and proved for the specific detection of AIF_Δ101_ (Figure 1A). The progressive reduction of electrophoretic mobility in AIF_Δ101_> AIF_Δ101_:H2AX > AIF_Δ101_:CypA > AIF_Δ101_:H2AX:CypA samples correlated well with the expected ^app^MWs of these assemblies (83, 85, and 101 kDa, respectively). Furthermore, analysis by a second SDS-PAGE of the band containing the degradosome mixture evidenced comigration of the three proteins in CN-PAGE (Figure   1A). The stability and stoichiometry of this degradosome assembly was further confirmed by size-exclusion chromatography. The elution volume for AIF_Δ101_ decreased when mixed with CypA, H2AX or both at the same time (Figure 1B–D, Supporting Information (SI) Appendix), confirming formation of the corresponding stable complexes whose ^app^MWs are summarized in Table S2. The multimeric state of these mixtures was also analyzed at the single molecule level using atomic force microscopy (AFM) (Figure 1E, Figures S1 and S2, and SI Appendix). Imaging of samples containing AIF_Δ101_ and either CypA or H2AX showed features compatible with heterodimeric complexes with similar angles between the two protein components for both complexes (∼135°) (Figure S1D and E). Moreover, the greater population of hetero-dimers for CypA than for H2AX (55% vs. 30%, respectively; Table S3) suggested that the AIF_Δ101_:CypA interaction was the most stable. Lastly, imaging of mixtures containing the three degradosome protein components produced features consistent with the simultaneous detection of monomers (25%), heterodimers (43%), and heterotrimers (degradosome, 32%), with the last two showing elongated arrangements (Figure 1E and Figure S2, Table S3). Altogether, these data evidenced for the first time the ability of AIF for simultaneous interplay with these two nuclear proteins. Finally, we simulated energetically optimized degradosome ensembles using experimental information regarding interaction surfaces (Figure 1F, Figures S3 and S4, and SI Appendix). The constructed models agree well with elongated AFM topological morphologies and demonstrated certain flexibility at the AIF_Δ101_:CypA interplay surface. Both features might be of relevance to promote larger interaction areas with the double-stranded DNA (dsDNA) upon formation of the DNA–degradosome complex, being therefore, more efficient to exert the required endonuclease activity.

### DNA–degradosome assembly and cooperativity effects

**Fig. 2.**
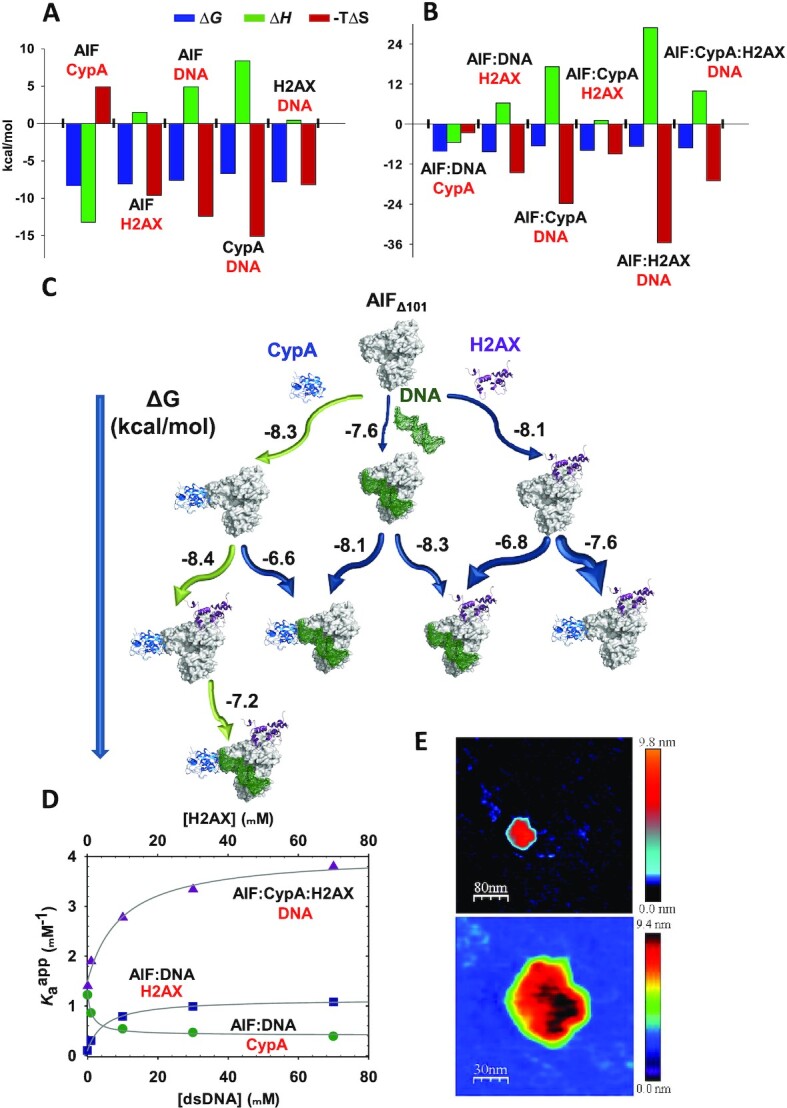
Binding mechanism upon degradosome assembly. Thermodynamic dissection of binary (A) and ternary (B) interactions as assayed from experimental ITC measurements shown in Figures S5 and S6. Gibbs energy (ΔG), enthalpy (ΔH), and entropy (-TΔS) contributions to the binding are represented in blue, green, and red bars, respectively. (C) Gibbs free energy flowchart for the degradosome assembly routes. The diagram summarizes the Gibbs energy of the interactions of AIF_Δ101_ with the different degradosome components as derived by ITC data in Figures S5 and S6. CypA (PDB: 3K0M) and H2AX (PDB: 6K1K chain C) are shown as cartoon colored in blue metallic and dark raspberry, respectively, AIF (PDB: 4BV6) is represented as gray surface and the dsDNA molecule (3′-GGT TAG TTA TGC GCG-5′, simulated through SCFBio tool (http://www.scfbio-iitd.res.in/software/drugdesign/bdna.jsp) is shown as dark green mesh. The length of the arrows is proportional to the ΔG for each interaction (values in kcal mol^−1^ are indicated in numbers), and their thickness are representative of the fraction of AIF_Δ101_ binding the titrating ligand. (D) Dependence of apparent association constants on the dsDNA concentration for the binding of CypA (*K_a_^app,CypA^*, green circles) and H2AX (*K_a_^app,H2AX^*, blue squares) to AIF_Δ101_:dsDNA; and dependence of apparent association constants on the H2AX concentration for the binding of dsDNA to AIF_Δ101_:CypA:H2AX (*K_a_^app,dsDNA^*, purple triangles). Data were fitted to Eq. (S2) and the fits represented by lines. All experiments were performed in 50 mM potassium phosphate, pH 7.4, at 15°C, or at 25°C when evaluating interactions involving CypA. (E) Representative AFM images of AIF_Δ101_:CypA:H2AX:dsDNA assemblies. The top image shows increased contrast to facilitate the observation of the dsDNA strands, diffused in the liquid, in dark blue. The bottom image shows a zoom of the assembly. In panels (A), (B), and (D) labels for single proteins and mixtures in the calorimetric cell are shown in black, while labels for the titrating ligands are in red.

The thermodynamics of interaction within binary complexes was then evaluated by isothermal titration calorimetry (ITC) (Figure 2A and Figure S5, SI Appendix and Table S4). The interaction of AIF_Δ101_ with CypA was enthalpically driven, in contrast to those with either H2AX or dsDNA. Nevertheless, all complexes displayed a moderate to significant affinity. The interaction of AIF with its protein nuclear partners was slightly stronger than with dsDNA, being CypA, the degradosome component that showed the lowest affinity for dsDNA under the assayed conditions. Moreover, the AIF_Δ101_:CypA binary association yielded the most favorable interaction, in correspondence with their suggested cotranslocation to the nucleus that supports the formation of the mentioned complex prior to binding either H2AX or dsDNA (7, 13).

Subsequently, we moved onto the determination of the binding contributions for the formation of ternary and quaternary assemblies (Figure 2B and Figure S6, Table S4). All interactions coursed with moderate affinity. In general terms, the interactions were entropically guided with an unfavorable enthalpic contribution, with the exception of the titrations of AIF_Δ101_:dsDNA and AIF_Δ101_:H2AX with CypA, which were enthalpically driven. A flowchart diagram of all assessed complexes with their corresponding free energies is presented in Figure 2(C). Noteworthy, the most favorable ternary interaction observed was that of AIF_Δ101_:CypA against H2AX (Table S4). Hence, the most favorable sequential mechanism of formation of the degradosome might be binding of CypA to AIF_Δ101_, followed by that of H2AX to AIF_Δ101_:CypA, and finishing with binding of dsDNA to AIF_Δ101_:CypA:H2AX (Figure 2C). Additionally, cooperativity effects within ternary and quaternary complexes were assessed (Figure 2D, Table 1 and Table S4). AIF_Δ101_ and CypA appeared to avidly compete against each other to interact with dsDNA (negative cooperativity), whereas the interaction of AIF_Δ101_ with dsDNA or CypA resulted significantly enhanced in the presence of H2AX (positive cooperativity). The degradosome complex, AIF_Δ101_:CypA:H2AX, happened to behave similarly to the latter, portraying a considerable positive cooperativity that resulted in an increased affinity for dsDNA as the concentration of H2AX became greater. Moreover, the sequential interaction of H2AX to a preformed AIF_Δ101_:CypA complex and, subsequently, of dsDNA to the resulting AIF_Δ101_:CypA:H2AX complex led to the greatest retention in electrophoretic–mobility–shift assays (EMSA) (Figure S7 and SI Appendix), confirming the above proposed sequential mechanism from ITC data as the main contributor to form the DNA–degradosome complex.

**Table 1.** Cooperativity coefficients (α) for the binding of CypA, H2AX, and dsDNA to AIF_Δ101_.

**Binary mixture in calorimetric cell**	**Titrating ligand**	α	***K*_a_^ligand^ (µM^−1^)**	***K*_d_^ligand^ (µM)**	***K*_a_^dsDNA^ (µM^−1^)**	***K*_d_^dsDNA^ (µM)**
AIF_Δ__101_:dsDNA	CypA	0.3	1.2	0.8	0.8	0.7
AIF_Δ__101_:dsDNA	H2AX	2.7	1.5	0.7	0.1	9.8
**Ternary mixture in calorimetric cell**	**Titrating ligand**	**α**	***K*_a_^ligand^ (µM^−^^1^)**	***K*_d_^ligand^ (µM)**	***K*_a_^H2AX^ (µM^−^^1^)**	***K*_d_^H2AX^ (µM)**
AIF_Δ__101_:CypA:H2AX	dsDNA	9.7	0.11	9.1	0.2	5.0

ITC assays were performed at 15°C, or at 25°C when evaluating interactions with CypA, in 50 mM potassium phosphate, pH 7.4. α represents the heterotropic cooperativity coefficient of the ligand binding (i.e., influence of one ligand prebound to AIF on the interaction of a second ligand), *K*_a_^ligand^ and *K*_d_^ligand^ are the intrinsic association and dissociation constants for the titrating ligand upon binding to the AIF_Δ101_:dsDNA or the AIF_Δ101_:CypA:H2AX complex. α and *K*_a_ values estimated from the fit to Eq. (S2).

The interaction of dsDNA with each protein partner was further analyzed using AFM (Table S6 and Figure S8). Imaging profiles indicative of dsDNA interaction were detected for each isolated protein, with heights corresponding to protein monomeric features bound to dsDNA. Remarkably, AIF_Δ101_ appeared to induce simultaneously stretching and opening of the dsDNA strands (Figure S8A). Moreover, it displayed cooperativity in the binding, with several AIF_Δ101_ molecules clustering around the same DNA strand, as previously observed by transmission electron microscopy (8). Heterodimers of AIF_Δ101_:CypA and AIF_Δ101_:H2AX appeared tightly bound to dsDNA (Figure S8D and E, respectively). AIF_Δ101_:CypA also produced a stretching on the DNA strands that was hardly detected when DNA was bound to AIF_Δ101_:H2AX. Moreover, the percentages of protein–protein association modes remained similar to those in the absence of dsDNA (Tables S3 and S6). On the contrary, images of the degradosome in the presence of dsDNA (Figure 2E and S8F) showed that the percentage of heterotrimers increased by nearly 2-fold (from 32% up to 52%), with the vast majority attached to dsDNA.

### The source of nuclease activity within the degradosome: both AIF and CypA harbor it

In order to assess the source of the nuclease activity of the degradosome, a plasmid vector was incubated with pure CypA, or combined with either pure AIF_Δ101_ or pure H2AX, or with both simultaneously, in the presence of 1 mM Ca^2+^ and 1 mM Mg^2+^ (both ions are required for the stimulation of the reported CypA nuclease activity (23)) (Figure S9A). Having been reported that AIF may recruit nucleases such as CypA and the macrophage migration inhibitory factor (MIF) to induce chromatinolysis (13, 24), we expected CypA to be the only actor to cleave DNA within the degradosome. Indeed, degradation of dsDNA was observed in all samples containing CypA, while the presence of H2AX offered some sort of protection by hindering dsDNA degradation over time. Meanwhile, the presence of AIF_Δ101_ considerably enhanced the degradation of dsDNA by increasing its smear as previously described (13), even though a negative cooperativity effect in the AIF_Δ101_:CypA binding to dsDNA has been observed in this work. At this point, it was also worth to consider some previous evidences that pointed out to recombinant mouse AIF (mAIF) as able to mediate dsDNA nicking and linearizing activities together, reflected in a discrete genomic DNA smear (Figures

4, 5, and S10 in references 13, 20, and 19, respectively). To further ascertain this, we incubated the same plasmid vector with pure AIF_Δ101_ (Figure S9B). Surprisingly, AIF_Δ101_ proved able to efficiently degrade the plasmid DNA in a similar fashion to CypA, revealing active nuclease capacity (23). Thus, AIF_Δ101_ transformed supercoiled dsDNA into its open circular and, subsequently, linear forms prior to degradation to lower MW forms, proving that it possesses both nicking and linearizing activities by itself (Figure S9B). It is remarkable to mention that CN- and SDS-PAGE together with mass spectrometry assays verified the purity of the AIF_Δ101_ samples, discarding any adventitious nuclease contamination (SI Appendix and Figure S10).

**Fig. 3.**
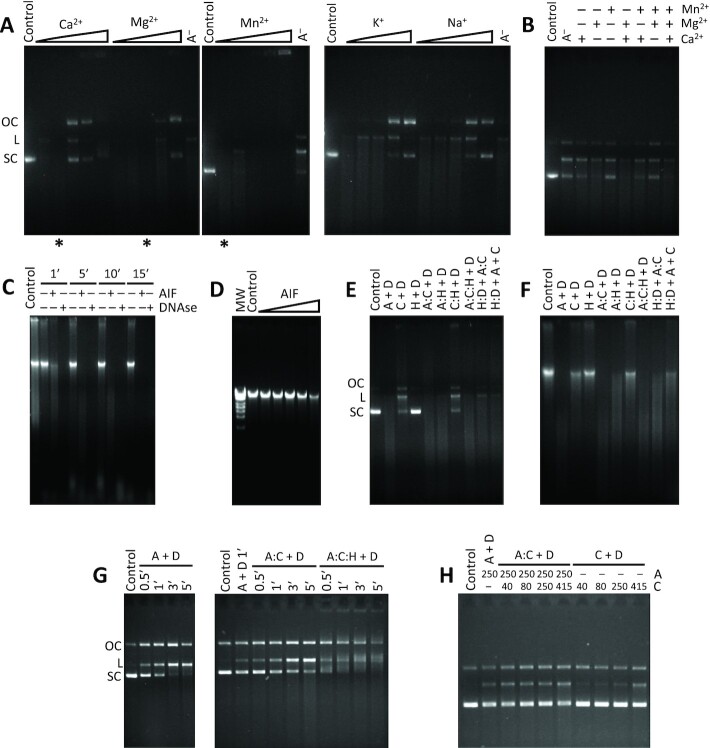
Influence of ions and protein partners on AIF nuclease activity. (A) Effect of different ions expected to promote (Ca^2+^, Mg^2+^, and Mn^2+^) or inhibit (K^+^ and Na^+^) the nuclease activity of AIF_Δ101_ against the plasmid DNA substrate. Increasing concentrations of ions (0.01, 0.1, 1, 10, and 100 mM) were evaluated. (B) Cooperative effect of promoting ions. Assays were carried out at the most optimal concentrations observed in A (0.01, 0.1, and 1 mM for Mn^2+^, Ca^2+^, and Mg^2+^, respectively), highlighted with asterisks at the bottom of each corresponding lane. (C) Effect of the incubation time (1, 5, 10, and 15 min) on AIF_Δ101_ nuclease activity against human genomic DNA. (D) Concentration-dependence of AIF_Δ101_ (0.025, 0.250, 2.5, 25, and 250 ng) in its nuclease activity against human genomic DNA. (E) and (F) Influence of CypA and H2AX—in various combinations and equimolecular concentrations—, on the AIF_Δ101_ nuclease activity against plasmid or genomic DNA substrates. (G) Time course of the influence of CypA and H2AX on the AIF_Δ101_ nuclease activity against the plasmid substrate. (H) Concentration-dependence effect of CypA (ratios 1:0.5, 1:1, 1:3, and 1:5 in micromolar) on AIF_Δ101_ nuclease activity. Samples in panels (G) and (H) were incubated at 30°C to facilitate visualization of changes. Unless otherwise stated double-stranded supercoiled pET-28a(+) plasmid substrate (250 ng) or human genomic DNA (500 ng) were mixed with purified AIF_Δ101_ (250 ng) (or 1 IU of DNAse in C) in 20 mM Tris/HCl, pH 8.0—supplemented with 0.1 mM CaCl_2_ and 1 mM MgCl_2_ in panels (C), (D), (E), and (F)—and incubated for 1 min at 37°C. Protein mixtures were preincubated for 15 min at 25°C. MW, molecular weight marker (GeneRuler High Range DNA Ladder; 10, 12, 14, 15, 17, 21, 25, and 50 kbp). Control, stands for plasmid or genomic DNA substrate alone. (A), (C), and (H), stand for AIF_Δ101_, CypA and H2AX, respectively. A^−^, stands for plasmid or genomic DNA incubated with AIF_Δ101_ in absence of ions (panels A and B). H:D + A:C, stands for H2AX plus DNA and AIF_Δ101_ plus CypA being separately preincubated and then mixed together. H:D + A + C, stands for H2AX and DNA being first preincubated together and subsequently mixed with separate samples of AIF_Δ101_ and CypA. OC, L, and SC, stand for open circular, linear, and supercoiled, respectively.

Subsequently, assessment of different enhancing divalent ions (Ca^2+^, Mg^2+^, and Mn^2+^) revealed that the human AIF_Δ101_ nuclease activity occurs in the absence of added ions, although it became optimally stimulated with 0.1 mM Ca^2+^ and 1 mM Mg^2+^ (Figure 3A and B). However, concentrations significantly below or above the optimal values resulted in an inhibitory effect on AIF nuclease activity, as similarly reported for other nucleases (25–27). Additionally, incubation in the presence of inhibiting monovalent ions (K^+^ and Na^+^) lead to a progressive decrease of the degradation profile, ultimately abolishing most of the nuclease activity (Figure 3A). Moreover, AIF nuclease activity remained evident on human genomic DNA from HeLa cells in a time- and concentration-dependent manner (Figure 3C and D). It initially digested the high MW forms (loss of DNA in the well) and transiently increased the lower MW forms until the dsDNA was completely degraded with no detection of the ladder pattern. Furthermore, we also confirmed nuclease activity in mAIF (Figure S11 and SI Appendix).

Having established the optimal conditions for the AIF nuclease activity, we studied the effect of the interplay among the degradosome components on DNA degradation. A set of nuclease assays were carried out by incubating the three proteins, either separately or in combination with plasmid and genomic DNA samples (Figure 3E and F). The latter were also analyzed with genomic DNA ScreenTape assays to determine the size of the remaining genomic DNA, the concentration of intact dsDNA and the DNA integrity quantified as DNA Integrity Number (DIN) (Table S7). Under the assayed conditions, AIF_Δ101_ turned out to be more efficient as nuclease than CypA on its own, showing a lower amount of remaining intact dsDNA (9% vs. 21%) and DIN (7.7 vs. 8.1). This is in agreement with the results obtained from the solution nuclease assays, where the CypA demonstrated activity on its own was estimated to be almost nine times lower than that of AIF (0.002 vs. 0.015 s^−1^) (Figure 3E). However, the CypA:AIF_Δ101_ combination hardly showed a slight increase in genomic DNA degradation (DIN 7.0 and 7% of intact dsDNA), possibly due to the negative cooperativity on CypA and DNA binding to AIF_Δ101_, but supporting their collaborative effect previously described in chromatinolysis (13). Interestingly, nuclease activities of AIF_Δ101_ and CypA induce the generation of ∼20 kbp and ∼50 kbp DNA fragments, respectively, similarly to what is observed during AIF-dependent cell death (1, 28). Moreover, addition of H2AX to AIF_Δ101_ has a protective effect on DNA degradation, giving DNA fragments of ∼50 kbp. For both, plasmid and genomic DNA substrates, the sample that yielded the greatest degradation was that of the AIF_Δ101_:CypA:H2AX degradosome (DIN 6.9 for genomic DNA), which was obtained through simultaneous incubation of the three proteins. The addition of AIF_Δ101_ and CypA to a preincubated H2AX:genomic DNA complex lead to the lowest DIN (6.6), but it resulted in twice the amount of remaining intact dsDNA (8% vs. 4%) when compared to the AIF_Δ101_:CypA:H2AX sample. Furthermore, a progressive increase in the time of incubation yielded a proportional increase in DNA degradation (coupled with a greater mobility shift), which was significantly higher for the degradosome (percentage of degraded DNA from 38% to 79% after 5 min) and AIF:CypA complex (7% to 35%) than for AIF on its own (6% to 28%) (Figure 3G). Increasing concentrations of CypA regarding AIF were additionally tested (ratios 1:0.5, 1:1, 1:3, and 1:5 in micromolar) at a set time of incubation, leading to a moderate increase in the percentage of degraded DNA (12% at ratio 1:0.5 to 18% at ratio 1:5) for the AIF:CypA complex that was significantly higher than that of AIF alone (10%) and even more so than those of CypA alone at the same concentrations (1% to 6%) (Figure 3H). All these results agree well with the proposed regulation of the chromatinolysis process via DNA–degradosome assembly during programmed necrosis induced by DNA alkylating agents (4).

The nuclease activity of the degradosome was also visualized by AFM in the presence of divalent cations. Figure S8H shows a representative image for AIF_Δ101_:CypA:H2AX:dsDNA when incubated with Ca^2+^ and Mg^2+^. Their presence contributed to overcome the threshold needed by the mica bound complex to degrade dsDNA into very small fragments, as can be observed in different parts of the zoomed images (Figure S8G and H). Interestingly, we also observed an equilibrium among open and compact morphologic states of the AIF _Δ101_:CypA:H2AX degradosome assemblies (Figure S8H and I). We can conclude that, in the absence of the mentioned ions the majority of the heterotrimers adopt closed forms (Figure 1E), which become more compact when bound to dsDNA (Figure 2E, S8F). However, the presence of ions favoring dsDNA fragmentation induces more elongated open heterotrimer conformations in equilibrium with the closed forms (Figure S8H and I). Finally, dsDNA preserved its integrity in the absence of nuclease activity (Figure S8J), while it underwent fragmentation when activity took place (Figure S8K).

### Key residues for AIF’s nuclease activity

**Fig. 4.**
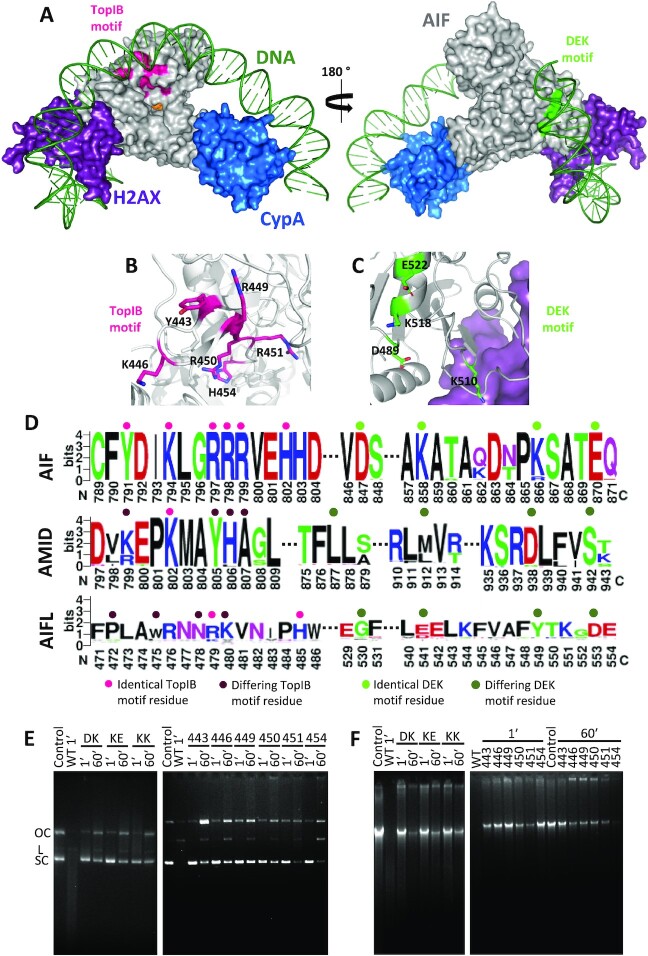
Potential nuclease domains in AIF and effect of TopIB and DEK mutations on its nuclease activity. (A) Model for the binding of dsDNA (green) to the energetically optimized degradosome assembly (color codes for proteins as in Figure 1F). The potential nuclease TopIB and DEK motifs appear colored in magenta and green respectively on the AIF surface. Environment of (B) TopIB and (C) DEK motifs highlighting relevant residues as sticks. (D) Sequence logo (https://weblogo.berkeley.edu/logo.cgi) for the conservation of identified TopIB and DEK motifs in human AIF in sequences of AIF family members (AIF, AIFL, and AMID) from different organisms. Key residues identical to those in human AIF TopIB and DEK motifs are marked respectively with a pink or green dot on top, while differing residues are marked with darker tones. While the motifs are significantly conserved within the AIF subfamily members, they do not extend to either AMID or AIFL proteins. AIF motifs, Yx_2_Kx_2_RRRx_2_H and Dx_9_Kx_7_Kx_3_E. AMID equivalents, kx_2_Kx_2_yha and lx_33_mx_23_dx_3_s. AIFL equivalents, px_2_wx_2_nRkx_4_H and gx_10_ex_7_yx_3_d. Capital letters indicate residues conserved across the different members, lower case letters stand for those not conserved. Further details in the Supplementary Material. Nuclease activity of different AIF_Δ101_ TopIB and DEK variants (E) on a double-stranded supercoiled pET-28a(+) plasmid (250 ng) and (F) on human genomic DNA (500 ng). dsDNA substrates were respectively mixed with the AIF_Δ101_ variants (250 ng) in 20 mM Tris/HCl, pH 8.0, 0.1 mM CaCl_2_ and 1 mM MgCl_2_. Samples were incubated for 1 and 60 min at 37°C. Control, stands for plasmid or genomic DNA substrate alone. OC, L, and SC stand, for open circular, linear, and supercoiled, respectively. Code for mutated residues in AIF_Δ101_ variants: 443, Y443A; 446, K446A; 449, R449A; 450, R450A; 451, R451A; 454, H454S; DK, D489A/K518A; KE, K518A/E522A; and KK, K510A/K518A.

Metal-ion-independent DNases make use of a conserved nucleophile residue to undergo catalysis, by means of an attack on the scissile phosphate of the nucleic acid that is aligned into optimal position by positively charged side chains nearby (29). One of such cases is that of the type IB topoisomerase (TopIB) family, whose active site is composed of two arginine residues, one lysine, one histidine and the nucleophile tyrosine. The DNA backbone is oriented and neutralized by the basic residues, with one conserved arginine hydrogen-bonded to the scissile phosphate and the tyrosine (29). AIF happens to present in its surface a sizeable positively charged pocket containing a motif that highly resembles that of the TopIB family (residues Y443, K446, R449, R450, R451, and H454) (Figure 4A and B). In fact, some of these residues belong to the so-called DNA-crown of AIF, the predicted DNA-binding site (10). Following the imaginary line drawn by the residues of the DNA-crown around the protein, another possible nuclease motif, a DEK motif, can be found close to the binding site of H2AX, composed of residues D489, E522, and K510 or K518 (Figure 4A and C). DEK motifs are conserved in many nucleases, including the recently identified PARP1-associate nuclease MIF (29, 30). Moreover, K510 and K518 residues were already shown to influence the interaction of AIF with dsDNA (10). Noticeably, these putative motifs are fully conserved in AIF sequences but not in the other two members of the AIF family, AMID and AIFL (Figure 4D), suggesting a common nuclease strategy for DNA degradation within the AIF subfamily (31). This strongly supports the relevance of these motifs in AIFs and in the different mechanisms during cell death among these three apoptotic effectors. To prove such hypothesis, we generated single-site mutants of all the aforementioned TopIB positions and double-site mutants for the potential DEK motif (namely: D489A/K518A, K518A/E522A, and K510A/K518A). All variants were purified to homogeneity and exhibited similar absorbance and circular dichroism spectra to the wild-type (WT), indicating that mutations did not significantly compromise neither the interaction with the cofactor nor protein folding (SI Appendix and Figure S12).

The nuclease activity of these variants was evaluated using both plasmid and human genomic DNA substrates (Figure 4E and F, and Table S7). Remarkably, all variants significantly impaired the nuclease activity of AIF_Δ101_, regardless of the substrate, and required at least 1 hour for the linearizing activity to become apparent (vs. less than 1 min for the WT protein). In addition, most variants produced a DNA retention effect not apparent for the WT under the assayed conditions (Figure 4E and F). EMSA assays (Figure S13) demonstrated that all variants yielded a considerably higher retention of the DNA substrate compared to the WT (with the sole exception of Y443A, for which it was only slightly greater) in agreement with the very subtle and localized electrostatic surface potential changes induced by the mutations (Figure S15). Moreover, variants D489A/K518A and K510A/K518A considerably increased affinity for dsDNA (*K*_d_ 0.5 vs. 2.9 µM for WT) (Table S8).

Regarding the impact of mutations on genomic DNA integrity (Table S7), K510A/K518A produced the lowest DIN (6.9), hinting that K510 might not belong to the potential DEK motif of AIF. In comparison, D489A/K518A and K518A/E522A produced much higher DIN values (8.4 and 8.3, respectively) and did not experience a shift in the size of the genomic DNA peak (>60 kbp for both, vs. 42 kbp for K510A/K518A). Consequently, the potential DEK motif of AIF may be made up of residues D489, K518, and E522 (SI Appendix and Figure S14A). On the other hand, all variants of the potential TopIB motif gave rise to significantly high DIN values (>8.7) and no shift in the size of the DNA peak (>60 kbp for all except R451A, whose peak was at 58 kbp). The Y443A, K446A, and R449A variants yielded the highest DIN values (>9.6), in agreement with the expected critical roles for these residues during the nucleophilic attack on dsDNA (SI Appendix and Figure S14B). Altogether, these results confirm the existence of TopIB and DEK motifs on AIF, granting the protein the potential ability to in vivo degrade dsDNA. Such hypothesis is further supported by the fact that mutants at these motifs were inefficient apoptosis sensitizers or unable to induce cell death in cell-free systems as well as in intact transfected cells (10).

### Concluding remarks and unresolved issues

Since AIF was discovered as a mitochondrial flavoprotein able to induce chromatin condensation in purified nuclei (1), a tremendous number of studies have identified it as one of the main effectors of caspase-independent cell death. However, the exact molecular mechanism through which AIF provokes chromatin remodeling, large-scale DNA fragmentation, and DNA loss remains unknown. Moreover, AIF’s contribution to PCD and its interactions with prodeath partner executers appear to be highly dependent on the organism, cell type and kind of apoptotic stimulus (9, 28, 32–34).

In this work, we have delved into the mechanism of AIF’s proapoptotic action. The interaction of AIF with H2AX and CypA as a tool to regulate chromatinolysis was described in programmed necrosis activated by alkylating DNA agents (4). Here, we evidence for the first time the assembly of this degradosome complex at the molecular level. Moreover, we identify cooperative effects between AIF and H2AX, as well as among the three protein components of the degradosome to promote dsDNA binding and degradation. These in vitro results agree with the required synchronized presence of these three proteins in the nucleus to provoke DNA degradation (4). Alternatively, the nuclear AIF:CypA cotranslocation and cooperation was proposed in damaged neurons after hypoxia–ischemia to promote DNA degradation (13, 7). This model is also compatible with a high plasticity of AIF to interact with the different degradosome components, being the interaction with CypA the most favorable and seemingly the only one specific.

Interestingly, in the above caspase-independent PCD models, as well as in others described in the literature, the DNA-degrading capacity of AIF is associated with the recruitment of nucleases, such as CypA, MIF, or endonuclease G (13, 24, 35). However, the depletion of these nucleases resulted in lower levels of chromatinolysis and cell death induced by MNNG, while the depletion of AIF was an absolute requirement to block caspase-independent cell death (2). Our work breaks away from this paradigm, providing compelling evidence that AIF_Δ101_ is endowed with nuclease activity. Thus, here it is shown that AIF can act as an efficient nuclease to cleave genomic DNA into large fragments by itself or in cooperation with CypA. Moreover, mutation of key residues in its TopIB and DEK nuclease motifs markedly reduced its in vitro nuclease activity and even prevented cell death (10). Therefore, beyond being a platform protein or recruitment factor, AIF also behaves as an apoptotic nuclease. These findings open trails for further researches focused on the in vivo involvement of the AIF nuclease activity in dying cells during apoptosis, and on how it might modulate or coordinate to degrade chromosomal DNA in mammalian systems. This knowledge will be key to develop novel therapeutic strategies to treat or prevent diseases associated with the aberrant behavior of AIF.

## Supplementary Material

pgac312_Supplemental_File

## Data Availability

The data that support the findings of this study are available in this manuscript and the Supplementary Material.

## Notes

Patricia Ferreira

Classification: Biological Sciences, Biochemistry.
