# Supplementary material for: Beyond a platform protein for the degradosome assembly: The Apoptosis-Inducing Factor as an efficient nuclease involved in chromatinolysis
Source: PNAS Nexus. 2022 Dec 26;2(2):pgac312. doi: 10.1093/pnasnexus/pgac312 (PMC9944232; doi:10.1093/pnasnexus/pgac312)
Supplement: pgac312_Supplemental_File [file pgac312_supplemental_file.pdf]

SI Appendix

Beyond a platform protein for the degradosome assembly: The Apoptosis Inducing Factor as efficient nuclease involved in chromatinolysis

Nerea Novo[a,b], Silvia Romero-Tamayo[a,b], Carlos Marcuello[c,d], Sergio Boneta[a], Irene Blasco-Machin[a], Adrián Velázquez-Campoy[a,b,e,f], Raquel Villanueva[a,b], Raquel Moreno-Loshuertos[a], Anabel Lostao[c,d,g], Milagros Medina[a,b*] and Patricia Ferreira[a,b*].

[a]Departamento de Bioquímica y Biología Molecular y Celular, Facultad de Ciencias, Universidad de Zaragoza, Spain.

[b]Instituto de Biocomputación y Física de Sistemas Complejos, BIFI (GBsC-CSIC Joint Unit), Universidad de Zaragoza, Spain.

[c]Instituto de Nanociencia y Materiales de Aragón (INMA), CSIC-Universidad de Zaragoza, 5009 Zaragoza, Spain

[d]Laboratorio de Microscopas Avanzadas (LMA), Universidad de Zaragoza, 50018 Zaragoza, Spain

[e]Aragon Institute for Health Research (IIS Aragón), Zaragoza, Spain.

[f]Biomedical Research Networking Centre for Liver and Digestive Diseases (CIBERehd), Madrid, Spain

[g]Fundación ARAID, Aragón, Spain

Supplementary Materials and Methods

Overexpression and production of proteins

Constructs used to overexpress AIF$_{\Delta 101}$ proteins as well as CypA were previously reported in (1). The cDNA sequences encoding for Y443A, K446A, R449A, R450A, R451A, H454S, K510A/K518A, D489A/K518A and K518A/E522A AIF$_{\Delta 101}$ variants (UniProtKB O95831) were obtained by site-directed mutagenesis from GenScript®. Similarly, the cDNA encoding for H2AX was codon optimized for *E. coli* expression and synthetized with a cleavable N-terminal His$_6$-tag (CACCAT) followed by a cleavable recognition site for PreScission Plus protease by GenScript®. The encoding sequence was subcloned between the *Nco*I and *Nde*I

sites of the pET-28a(+) plasmid. $AIF_{\Delta 101}$ variants and CypA were produced and purified using Ni^{2+} affinity chromatography as previously described (1). For H2AX overexpression, *E. coli* cultures were grown at 37 ºC and 180 rpm in LB supplemented with 30 mg/L of kanamycin (Sigma-Aldrich). When cultures reached OD_{600nm} ~0.6, 1 mM IPTG was added to induce protein expression, and they were incubated for 3 additional hours. Cells were then harvested, resuspended in 50 mM Tris/HCl, pH 8.0, and lysed by sonication on ice. The cell lysate was centrifuged to remove debris, and subsequently mixed with Ni^{2+} IMAC Sepharose 6 Fast Flow gel (GE Healthcare) equilibrated in the aforementioned buffer supplemented with 0.4 M KCl and 4 mM imidazole. This mixture was incubated on a bidirectional rocker for 45 min at 4 ºC, and then packed into an empty column. All subsequent steps were performed at 4 ºC in 50 mM potassium phosphate, pH 7.4, 150 mM NaCl. The matrix was washed with 200 mM imidazole to discard contaminants, after which impure H2AX was eluted with 1 M of imidazole. Fractions were pooled and subsequently loaded to a PD–10 column (GE Healthcare) to remove the imidazole content, filtered and loaded into a 1–mL HisTrap™ High Performance column (Cytiva). The matrix was then washed with 350 mM imidazole and pure H2AX was finally eluted with 1 M imidazole. Fractions were pooled and the imidazole content was removed in a PD–10 column as before. In all cases protein purity was assessed by SDS-PAGE and molecular exclusion chromatography. Additionally, purity of $AIF_{\Delta 101}$ was further confirmed by mass spectrometry, discarding any adventitious nuclease contamination.

$AIF_{\Delta 101}$ concentration was determined using the previously reported ε_{451nm} of 13.77 $mM^{-1}\cdot cm^{-1}$ (2). To determine CypA and H2AX concentrations theoretical $\varepsilon_{280\ nm}$ values obtained from the ProtParam tool (ExPASy) were used (8.73 $mM^{-1}\cdot cm^{-1}$ and 5.96 $mM^{-1}\cdot cm^{-1}$, respectively). All proteins were stored in 50 mM potassium phosphate, pH 7.4, at -80 ºC.

Clear Native (CN) and 2D denaturing electrophoresis

CN gradient electrophoresis was first run at 80 V and 4 ºC for 25-30 min, and then the amperage was set to 12 mA/gel and the voltage limited to 300 V until the sample front reached the bottom of the gel (~120 min in total). For 2D analysis, the first-dimension lane was cut out from the gel and incubated for 1

hour at 25 ºC in 1% SDS and 1% β-mercaptoethanol. The supernatant was then run in a 15% second-dimension denaturing gel (2D SDS-PAGE) at 4 ºC and 30 V for 25-30 min, and after that the voltage was set in the 80-120 V range until the dye reached the bottom of the gel. Gels were electroblotted onto Hybond-P PVDF membranes (Amersham) and then probed with specific antibodies against AIF and His-tag for CN-PAGE and SDS-PAGE respectively. Detection of immunoreactive proteins was performed using HRP-conjugated secondary antibodies (Thermo Fisher Scientific). Signals were detected using the EZ-ECL Chemiluminescence Detection kit from HRP (Pierce™), and immunoblot images were obtained in an automated WB processor Amersham™ Imager 600 (GE Healthcare).

AFM imaging measurements

Samples of protein complexes were prepared by mixing AIF_{101} (0.5 µM) with CypA or/and H2AX in 1:1 molar ratios for 10 min at 4 ºC under mild stirring. These mixtures, as well as the free proteins, were also mixed with 0.05 ng/µl of the pET-28a(+) plasmid -linearized with EcoRI- to visualize protein binding to dsDNA. Final concentrations were chosen to ensure the observation of individual features and thus, to facilitate complex identification and further analysis (3). All samples were prepared in PBS pH 7.0 (Thermo Scientific). For dsDNA degradation assays, mixtures were prepared in the presence of 5 mM $CaCl_2$ and 5 mM $MgCl_2$ to stimulate nucleolytic activity. Samples were incubated on fresh cleaved mica pieces (Electron Microscopy Sciences) for 10 min at room temperature to achieve molecular immobilization. In the case of samples involving dsDNA, a pre-treatment with 200 mM $MgCl_2$ was applied for 2 min to favor attachment of its strands to the negative hydroxyl groups at the mica surface (4). Then, the mica pieces were washed three times with the same buffer to prevent non-desirable interactions among free biomolecules and the AFM tip, which might disturb image acquisition. At least 10 representative images from 10 different areas of 200 x 200 nm and 400 x 400 nm were analyzed for protein and protein-DNA samples, respectively. The resolution of all AFM images was at least of 512 x 512 pixels and the acquisition rate was defined at 0.5 Hz. Estimation of percentages and their associated errors were calculated as previously described for the different

association states (5). Raw AFM images were analyzed using the WSxM free software (6).

ITC measurements

dsDNA samples (0.5 mM) for ITC titrations were prepared by annealing 1 mM solutions of the forward and reverse ssDNA 15-bp oligonucleotides (5′- GGT TAG TTA TGC GCG -3′ and 5′- CGC GCA TAA CTA ACC -3′; synthetized by Integrated DNA Technologies) upon incubation at 99 ºC for 1 min, followed by a 3-hour temperature gradient from 95 to 25 ºC (decreasing 1 ºC every 3 min). This length of dsDNA was designed as an appropriate model for a one to one interaction, since dsDNA was predicted to interact with AIF through no more than 12 bp (7).

To evaluate the buffer independent binding enthalpy (ΔH^{o}), titrations were carried out using a set of buffers with different ionization enthalpies (**Table S1**) (8).

Table S1. Enthalpies of ionization of used buffers.

Buffer	Reaction	pK_a	ΔH_{ion} (kcal/mol)
**Phosphate**	$H_2PO_4^- = H^+ + HPO_4^{2-}$	7.19	0.86
**HEPES**	$HL^{\pm} = H^+ + L^-$ (HL= $C_8H_{18}N_2O_4S$)	7.56	4.80
**MOPS**	$HL^{\pm} = H^+ + L^-$ (HL= $C_7H_{15}NO_4S$)	7.18	5.04
**TES**	$HL^{\pm} = H^+ + L^-$ (HL= $C_6H_{15}NO_6S$)	7.76	7.80
**TRIS/HCl**	$HL^+ = H^+ + L$ (HL= $C_4H_{11}NO_3$)	8.07	11.51

Values at 298.15 K and 0.1 MPa. Data from (Goldberg, Kishore, & Lennen, 2002).

Fitting of the dependence of the observed binding enthalpy (ΔH) on the buffer ionization enthalpy (ΔH_{ion}) to the equation:

$$\Delta H = \Delta H^{\circ} + n_{H^+} \cdot \Delta H_{ion} \text{ (Eq. S1).}$$

allowed determination of ΔH^{o} and the number of protons exchanged between the complex components and the bulk solution (n_{H^+}). If n_{H^+} is positive, the complex

formation occurs with capture of protons from the solvent, while a negative value indicates releasing of protons to the solvent (9).

To determine the binding cooperativity effects, one component of the preformed binary or ternary complex was varied from 1 to 8–fold, while keeping the other components fixed. For that, sets of experiments were performed by locating mixtures containing 10 µM $AIF_{\Delta 101}$ and 10, 20, 40 or 80 µM dsDNA into the calorimetric sample cell. These mixtures were then titrated with either CypA or H2AX (100 µM in the syringe) and analyzed as binary titrations, thus estimating the apparent binding parameters for CypA or H2AX binding to the $AIF_{\Delta 101}$:dsDNA complex. For ternary mixtures, sample cells containing 10 µM $AIF_{\Delta 101}$:CypA complex (1:1) and 10, 20, 40 or 80 µM H2AX were separately titrated with dsDNA (100 µM in the syringe). These experiments allowed to determine the apparent association constant for the titrating-ligand to $AIF_{\Delta 101}$:CypA, $AIF_{\Delta 101}$:H2AX or $AIF_{\Delta 101}$:CypA:H2AX complexes ($K_a^{app,titrating\text{-}ligand}$). These $K_a^{app,titrating\text{-}ligand}$ values were then fitted to equation S2, which describes their dependency as a function of the concentration of the varying cell-ligand pre-bound to $AIF_{\Delta 101}$ or $AIF_{\Delta 101}$:CypA complex and of the cooperativity constant (α) (10):

$$K_a^{app,titrating-ligand} = K_a^{titrating-ligand} \cdot \frac{1 + \alpha K_a^{cell-ligand}.[cell - ligand]}{1 + K_a^{cell-ligand}.[cell - ligand]} \text{ (Eq.S2).}$$

where $K_a^{app,titrating\text{-}ligand}$ is the association constant for the ligand in the syringe at each concentration of the cell-ligand, $K_a^{cell\text{-}ligand}$ is the association constant for the ligand in the cell in binary complex with $AIF_{\Delta 101}$ or in ternary complex with $AIF_{\Delta 101}$:CypA, and [cell-ligand] is the concentration of free cell-ligand in the calorimetric cell.

Quantification of DNA degradation by densitometry

Densitometry was employed to quantitate DNA degradation from the solution nuclease assays. Agarose gels stained with ethidium bromide were scanned with a Gel Doc EQ (Bio-Rad) system and subsequently quantitated using the Quantity One (Bio-Rad) software. To calculate the percentage of non–degraded DNA, the area comprising each peak was delimited and the intensity of signal within it was measured. Background noise was calculated likewise, delimiting an area of identical dimensions in the same well but with no peak, and subsequently subtracted from the intensity of samples and control signals. The measured

intensity of the control (DNA without nuclease) was considered to be 100%. To determine the relative rate of DNA degradation, the estimated amount of degraded DNA (ng) was divided by the time of degradation (seconds) and the amount of AIF (ng).

Docking and molecular dynamics (MD) simulations to build molecular degradosome and dsDNA-degradosome models

Rigid body docking (pyDockWeb server (11)) and molecular dynamics (MD) simulations (GROMACS 2018.4. (12)) were used to produce energetically optimized AIF:CypA:H2AX degradosome models. The initial AIF:CypA model was built using the coordinates from PDB:3K0M for CypA and PDB:4BV6, after modelling the missing C-loop segment, for oxidized AIF (1). Restrictions for the regions 55-69 of CypA and/or 370-394 of AIF, experimentally reported as involved at the interaction surface, were used in the classification of docking energies (13). The AIF:CypA lowest energy structure was submitted to MD. Subsequently, H2AX was docked onto the AIF:CypA complex using as receptor different frames obtained in AIF:CypA MD simulations and the PDB:6K1K chain C of H2AX. The 544-560 region of AIF was used to restrict the interacting region during classification of the docked poses, since it has also been experimentally mapped (14). MD was then performed on the best 3 poses. For MD simulations, complexes were protonated to pH 7.0 using PROPKA (15). Protein and FAD parameters were generated using respectively pdb2gmx and Gaussian 09 (16). The system was solvated with a TIP3P water model in a triclinic box and neutralized by adding ions. Steepest descent minimisation was performed to avoid close contacts. Equilibration was first conducted under a 500 ps NVT ensemble and then under a 500 ps NPT ensemble, with atoms of protein and ligand restrained with a 1000 $kJ.mol^{-1}.nm^{-1}$ harmonic potential. 50 ns NPT simulations at 310 K with unrestrained positions were then performed. Data were analyzed using GROMACS 2018.4. package tools (12), VMD (17), PyMol (18) and the graphic software Origin (OriginLab, USA).

The HADDOCK 2.4 web server (19) was used to explore the preferred docking sites for unspecific dsDNA of 15 to 20 bp sequences on CypA, H2AX and AIF, when free and when forming the degradosome. Residues of either the TopIB or DEK motives were used to restrict the dsDNA AIF interaction site, while no

restrictions were imposed for dsDNA docking on H2AX or CypA. Residues showing in these dockings a high propensity to interact with dsDNA were then used to model the wrap of a dsDNA 80 bp strand onto the above built degradosome model by using Graphite-LifeExplorer (20).

Sequence logo

To create the sequence logos, the amino acid sequence of each of the three proteins of the human AIF superfamily were separately downloaded from GenBank of NCBI (references NP_004199.1, NP_001185625.1 and NP_653305.1 for AIF, AMID (apoptosis-inducing factor-homologous mitochondrion-associated inducer of death) and AIFL (apoptosis-inducing factor-like), respectively) (21). An individual protein BLAST alignment (https://blast.ncbi.nlm.nih.gov) was subsequently performed for each target sequence using default parameters, and the first 500 sequences were selected. Each target was~~,~~ then globally aligned with its selected sequences using the ClustalW algorithm in the MEGA-X software (https://www.megasoftware.net) and a WebLogo was constructed with the resulting alignment (https://weblogo.berkeley.edu/logo.cgi).

Supplementary Results

1. Visualizing the degradosome assembly at the molecular level

1.1 Analysis of degradosome assembly by size exclusion chromatography

In the size exclusion chromatography analysis, binary mixtures containing $AIF_{\Delta 101}$ and either CypA or H2AX eluted mainly as new broad peaks of lower exclusion volume than that of free $AIF_{\Delta 101}$. **Table S2** summarizes the elution volumes for these peaks, showing that they correspond to assemblies with ^{app}MW of 53 and 50 kDa that respectively match those for hetero-dimers of $AIF_{\Delta 101}$:CypA and $AIF_{\Delta 101}$:H2AX. In the case of ternary mixtures, the deconvolution profile of the principal elution peak suggests two populations of similar proportions with ^{app}MWs of 57 and 47 kDa. The first matches well with the expected MW for the degradosome assembly, whereas the second corresponds to monomeric free $AIF_{\Delta 101}$.

Table S2. Quaternary assemblies of $AIF_{\Delta 101}$ with its apoptotic partners detected by gel filtration chromatography.

Samples	^{app}MW (kDa)				Complex assembly (stoichiometry)
	Peak 1	Peak 2	Peak 3	Peak 4	
**$AIF_{\Delta 101}$**		47			Monomer
**CypA**			13		Monomer
**H2AX**				5	Monomer
**$AIF_{\Delta 101}$:CypA**	53		13		Hetero-Dimer (1:1)
**$AIF_{\Delta 101}$:H2AX**	50			5	Hetero-Dimer (1:1)
**$AIF_{\Delta 101}$:CypA:H2AX**	57	47	14	nd	Hetero-Trimer (1:1:1)

Single free proteins and pre-formed mixtures of $AIF_{\Delta 101}$ with different partners (1:3 ratio) in 50 mM potassium phosphate, pH 7.4, were passed through a Sephadex S-200 column in the same buffer supplemented with 10 mM NaCl. ^{app}MWs of the elution peaks were obtained by Gaussian deconvolution of the gel filtration elution profiles (Figure 1 B-D). The table shows the calculated ^{app}MW for the principal component of each population. The column was calibrated with a set of proteins of known MW. nd: not detected.

1.2 Analysis of the degradosome assembly by AFM

When taking images of $AIF_{\Delta 101}$, CypA and H2AX by AFM (**Figure S1A-C**), their respective average heights of 6.3 ± 0.9 nm, 3.9 ± 0.6 nm and 4.6 ± 0.7 nm agreed well with the dimensions of their corresponding PDB structures, indicating they were visualized mostly as monomers (95-98%). Besides that, no features bigger than occasional homo-dimers were observed (**Table S3**). When similarly evaluating mixtures containing $AIF_{\Delta 101}$ and either CypA or H2AX, monomers corresponding well to the dimensions of isolated proteins were identified, but additional imaging features were also observed (**Figure S1D-E, Figure S2**). These features differed from the monomeric and homo-dimeric features of isolated proteins (**Figure S1A-C**), being compatible with the formation of stable hetero-dimeric complexes (**Figure S1D-E**). Lastly, the simultaneous incubation of the three protein components of the degradosome produced 25 % of monomers, 43 % of hetero-dimers and 32 % of hetero-trimers (**Table S3**). Moreover, overall height of the degradosome assemblies remained similar to those of the monomeric isolated features, suggesting that association of the three proteins takes place at the mica plane.

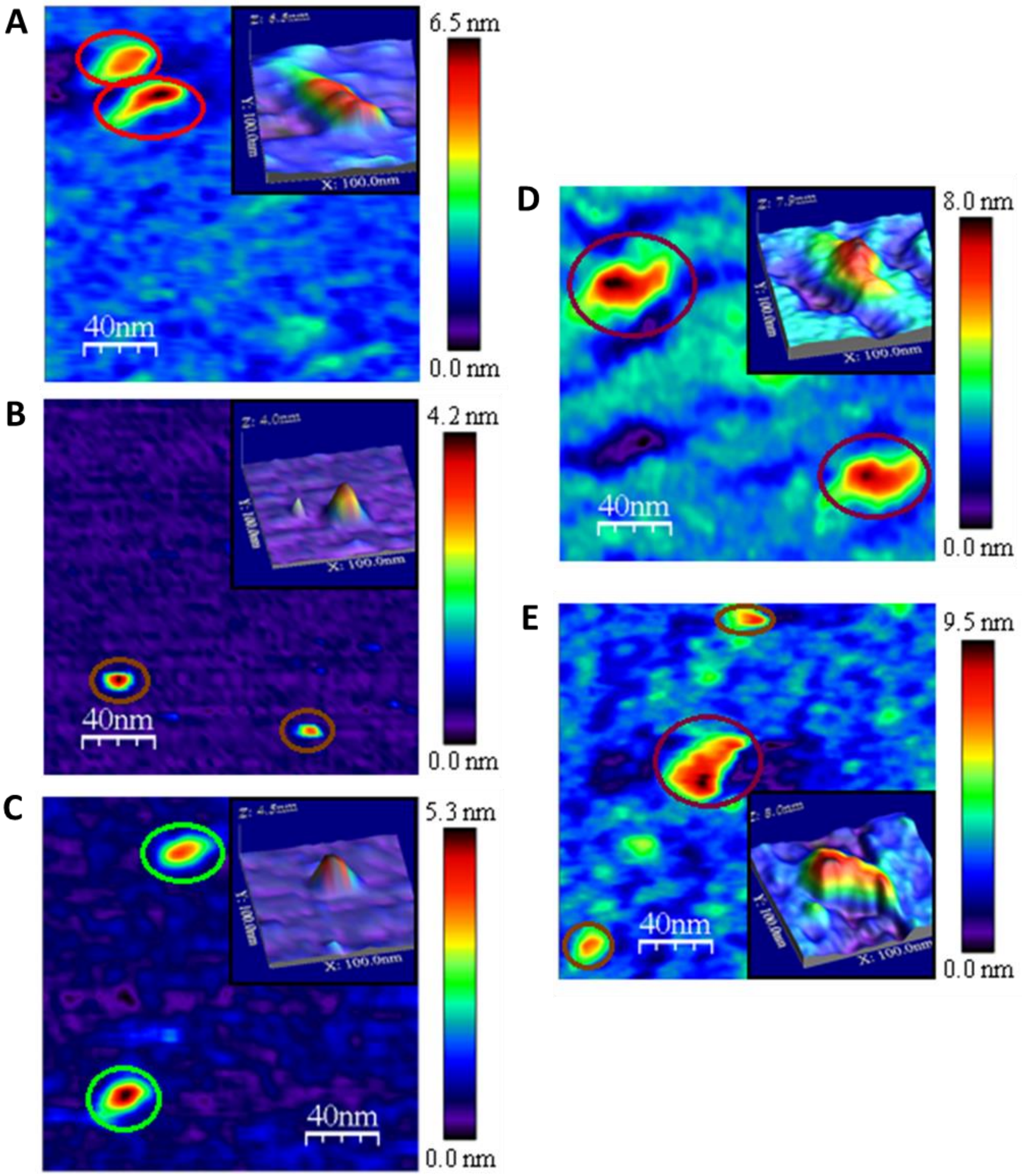

Figure S1. AFM topography of protein components of the degradosome and of their binary assemblies. Imaging of **(A)** $AIF_{\Delta101}$, **(B)** CypA, **(C)** H2AX, **(D)** $AIF_{\Delta101}$:CypA and **(E)** $AIF_{\Delta101}$:H2AX samples. $AIF_{\Delta101}$ (0.5 µM) was incubated with either H2AX or CypA (1:1 ratio) for 10 min at 4 ºC in PBS, pH 7.0. Scan size was 200 nm x 200 nm. Red, brown and green circles indicate $AIF_{\Delta101}$, CypA and H2AX monomers, while hetero-dimers are highlighted in purple. The inset panels depict zooms of representative 3D AFM images of the studied monomers or binary complexes (scan size 100 nm x 100 nm).

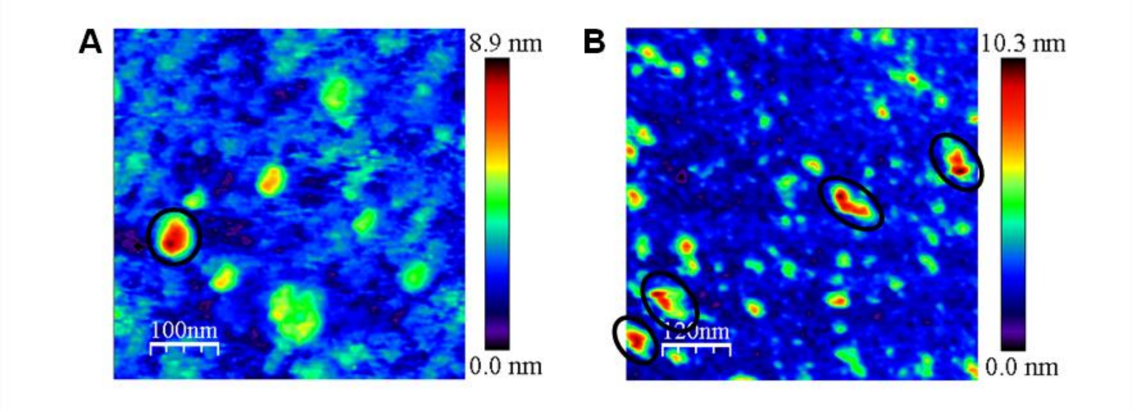

Figure S2. AFM topography field images of a sample of AIF$_{Δ101}$ incubated with CypA and H2AX (1:1:1 ratio). (A) AFM field image of the AIF$_{Δ101}$:CypA:H2AX assembly showed in Figure 1E. Scan size 500 nm x 500 nm. **(B)** AFM field image taken in a different area, where several AIF$_{Δ101}$:CypA:H2AX assemblies can be observed. Scan size 600 nm x 600 nm. AIF$_{Δ101}$:CypA:H2AX complexes rounded in black.

Table S3. Distribution of quaternary species identified by AFM imaging of AIF$_{Δ101}$, CypA, and H2AX, as well as of their binary and ternary complexes.

Sample mixtures	Monomers (%)	Hetero-Dimers (%)[a]	Hetero-Trimers (%)
**AIF$_{Δ101}$**	98	2	-
**CypA**	95	5	-
**H2AX**	95	5	-
**AIF$_{Δ101}$:CypA**	45	55	-
**AIF$_{Δ101}$:H2AX**	70	30	-
**AIF$_{Δ101}$:CypA:H2AX**	25	43	32

Protein samples in PBS, pH 7.0. Mixtures containing 0.5 μM of each indicated protein were pre-incubated before AFM imaging. Percentages are referred to the total number of protein molecules analyzed. Error is within 5-10 % of the percentage value. [a]In samples containing a single protein, values correspond to homo-dimers.

1.3 Structural modelling of the degradosome assembly

A sequential routine of rigid body docking and MD simulations was followed to produce energetically optimized assembles, first for the AIF:CypA binary association, and then for the AIF:CypA:H2AX degradosome. Molecular visualization of docking poses and frames of equilibrated MD trajectories, as well as analysis of equilibrated RMSF values for CypA/H2AX Cαs when aligning AIF position, inform of the conformational and flexibility changes within the interacting proteins (**Figure S3**). In both organizations, CypA regions including residues 55-60, 118-121 and 140-150 deviated less regarding AIF, whereas larger displacements were observed at other CypA positions. This suggests that whereas the above indicated regions maintain the AIF:CypA association, the pivotal rotation of CypA onto the AIF interaction surface is produced. CypA hot spot residues for binding to AIF particularly include the 58-PGF-60 hydrophobic loop and the R148 side chain, which stay at the interaction surface independently on CypA rotation (**Figure S4**). Noticeably, the CypA PGF region and W121 are reported as relevant for its peptidylprolyl isomerase activity, and cis/trans isomerization of prolines, with key roles in immunity and viral infection. In addition, F60 and W121 also contribute to the stacking of CypA to other proteins or peptides, such as the HIV-1 capsid (22), alpha-synuclein (23), the CrkII adaptor protein influencing cell motility and invasion (24) or cyclosporin A and derivative immune-suppressive drugs (25, 26). As expected, the AIF surface binding CypA is mainly contributed by the antiparallel β-sheet 370-390, being residue V374 a particular hot spot at its central site. Simulations also point to the 272-276 residues, particularly the E276 side-chain, as being involved in CypA binding (**Figure S4**). On its side, after MD equilibration of the degradosome assemblies, H2AX keeps mainly bound at the AIF C-loop Pro-rich region that includes a PST motif reported as chosen by this histone for binding to other partners (27). This motif remains as AIF's most flexible region even when H2AX is bound, since the dynamic deformation of the H2AX central α2-helix allows to accommodate its dock. Altogether simulations envisage that AIF acts as a docking platform to promote localization of CypA and H2AX, while allowing overall conformational orientation and flexibility. As a consequence, it is envisaged that the degradosome will be able to evolve in conformation upon interaction with other

biomolecules, as for example DNA, to facilitate their association as well as subsequent processes involved in chromatin condensation and DNA degradation.

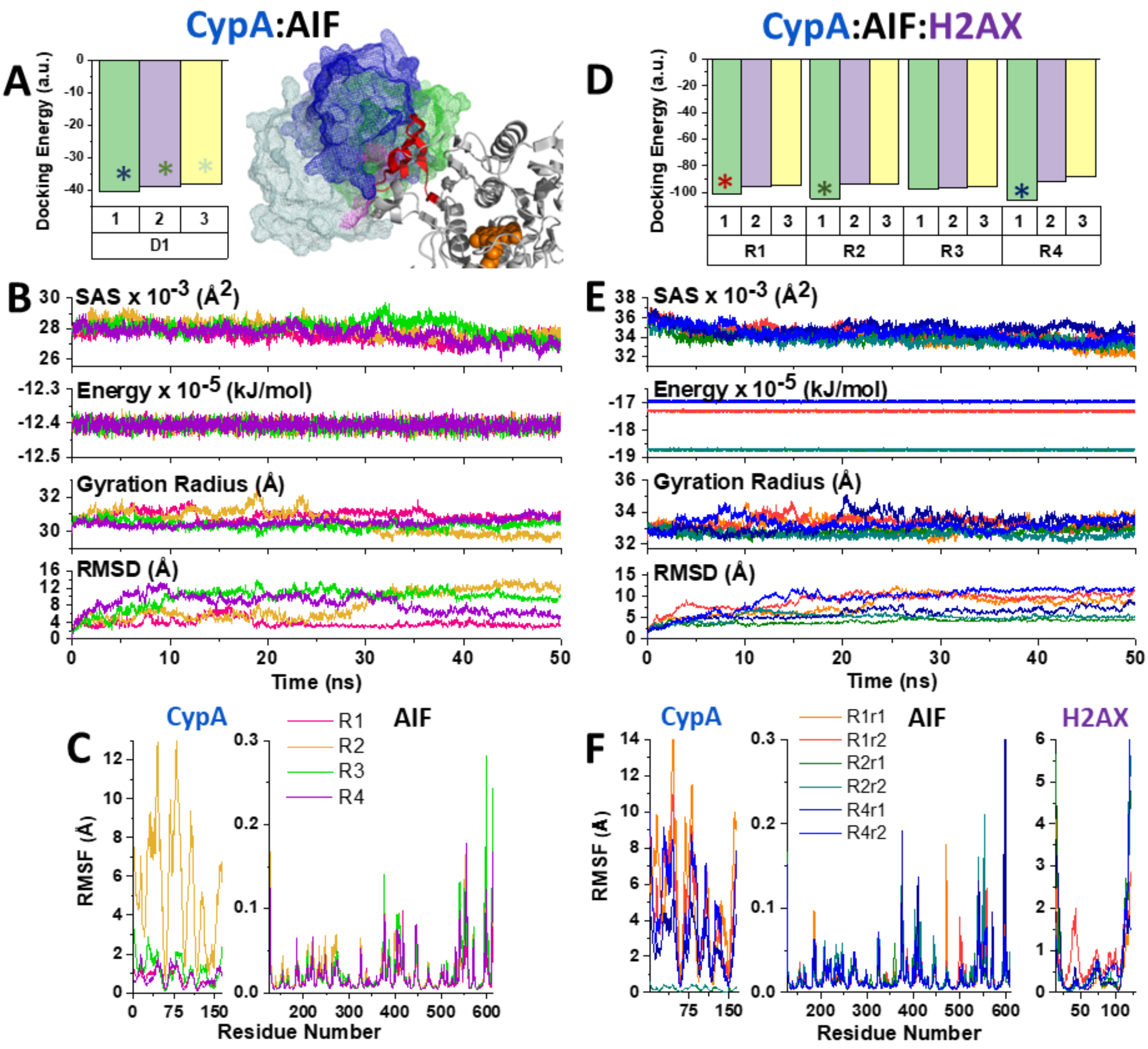

Figure S3. Modelling energetically optimized (eo) degradosome assemblies. (A) Docking energies (left) and relative disposition (right) for the three best poses for the docking of CypA onto AIF as the receptor. Best three CypA poses are shown in mesh and respectively colored in metal blue, green and pale green, while AIF is shown as grey cartoon with FAD as orange spheres. CypA and AIF regions used to restrict docking poses are highlighted in magenta and red respectively. **(B)** SAS, energy, radius of gyration and RMSD (Cα) trajectories showing equilibration along the AIF:CypA MD production. **(C)** RMSF (Cα) along MD for the proteins making the AIF:CypA complex. **(D)** Energies for the three best poses for the docking of H2AX to AIF:CypA$_{eo}$ models as receptors. Docking was performed for two selected frames of each of the four MD replicates of the selected AIF:CypA$_{eo}$ docking poses, but for clarity energy data are only shown for one frame in each replicate. Asterisks correspond to the three selected poses for subsequent energy optimization by MD. **(E)** SAS, energy, radius of gyration and RMSD (Cα) showing equilibration along the AIF:CypA:H2AX MD

production. **(F)** RMSF (Cα) along MD for the three proteins making the degradosome complex. In (E) and (F) data from the two MD replicates for each of the three selected docking poses are shown. Only frames upon reaching equilibrium (20-50 ns range of the MD production) were used for RMSFs calculation and they were locally aligned considering only the AIF chain.

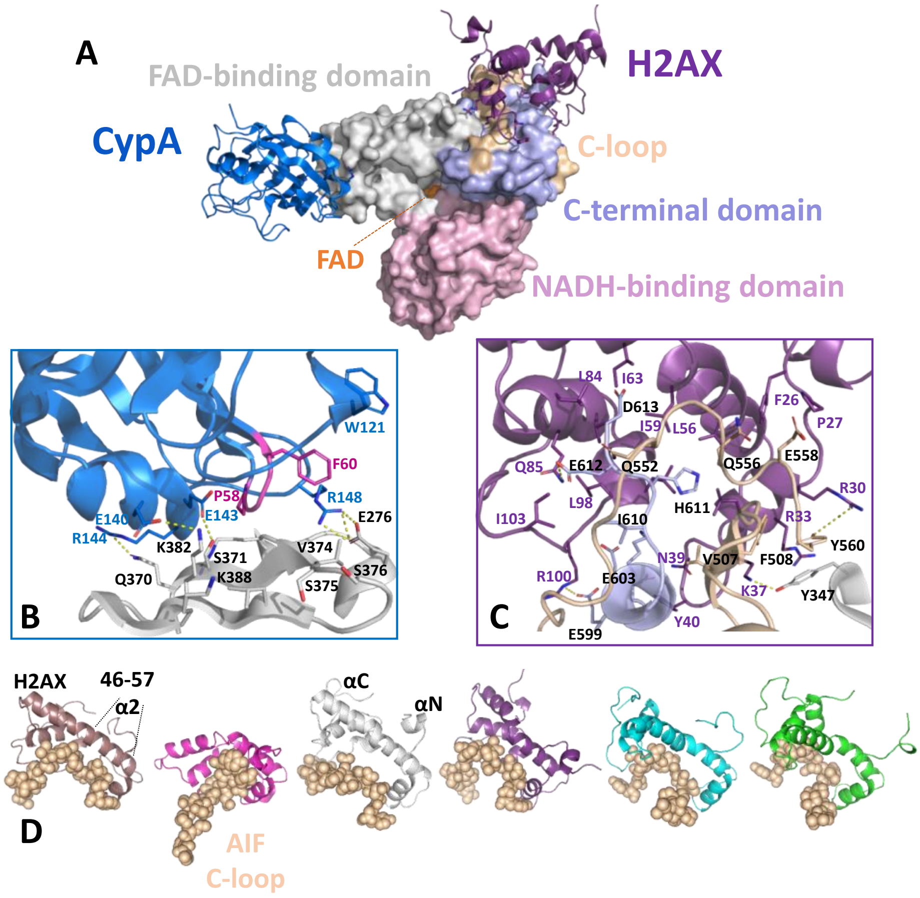

Figure S4. Energetically optimized model for the degradosome assembly. **(A)** Model selected from replicate R2r2 from Figure S3. AIF is shown as surface, with FAD-binding, NADH-binding and C-domains in grey, pink and light violet respectively, the C-loop highlighted in wheat and the FAD as orange spheres. CypA and H2AX are shown as metal blue and dark violet cartoons, respectively, and main residues implicated in the interaction with AIF as CPK colored sticks. Enlarged details of **(B)** AIF:CypA and **(C)** AIF:H2AX interaction surfaces, showing all proteins as cartoons and highlighting relevant residues as sticks. Some potential polar interactions are shown as yellow dashed lines. **(D)** Final frames for the six MD replicates of the degradosome complex showing the relative disposition of H2AX (at a different color for each replicate) and the AIF-Pro rich motif (residues 540-560, orange spheres). To highlight C-loop and H2AX conformational flexibility, H2AX models are shown locally aligned through the N-terminal of their α2-helix (residues 46-57).

2. DNA-degradosome assembly and cooperativity effects

2.1 Binary interactions between the components of the degradosome

Interaction within the binary complexes was evaluated among all of its components -namely, $AIF_{\Delta101}$, CypA, H2AX and dsDNA- through ITC. Binding isotherms were best fitted to a single binding site model with a K_d within the micromolar range for all binary complexes (**Figure S5 and Table S4**). Control ITCs were additionally carried out with the ligand in the syringe being titrated into the buffer, ensuring that there was no significant dilution effect on the heat change (**Figure S5A-C**, in red). Thermograms for the titration of $AIF_{\Delta101}$ with its protein partners, CypA and H2AX (**Figure S5A, B**), demonstrated that the interaction of $AIF_{\Delta101}$ with CypA is enthalpically guided, whereas binding of AIF to H2AX is driven by a favorable entropic contribution. This suggests that the $AIF_{\Delta101}$:CypA binary complex rises from specific polar interactions between both proteins, which is in agreement with previous results in the literature (1, 13). On the contrary, the interaction between $AIF_{\Delta101}$ and H2AX appears to lack such electrostatic specificity. Nevertheless, both binary complexes displayed a significant affinity between their components, with K_d values of 0.7 and 0.8 µM respectively (**Table S4**).

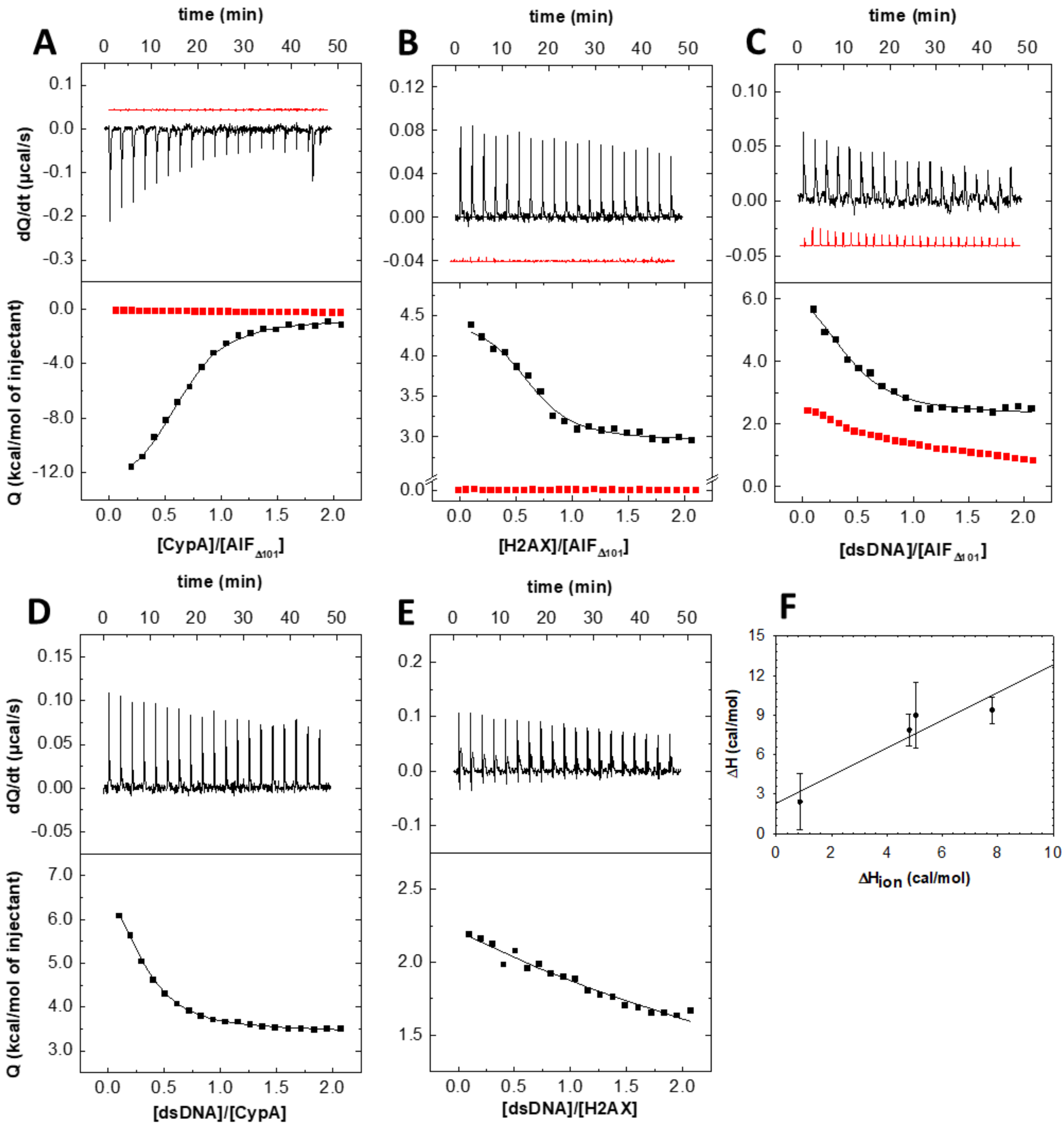

Figure S5. Isothermal calorimetric titrations for binary interactions of the degradosome components. ITC profiles for **(A)** AIF$_{\Delta101}$:CypA, **(B)** AIF$_{\Delta101}$:H2AX, **(C)** AIF$_{\Delta101}$:dsDNA, **(D)** CypA:dsDNA, and **(E)** H2AX:dsDNA complexes. Measurements were performed in 50 mM potassium phosphate, pH 7.4, at 15 °C, or at 25 ºC when evaluating interactions involving CypA. Panels A-C show control titrations (superimposed in red) for which ligands were put in the syringe and used to titrate buffer 50 mM potassium phosphate pH 7.4 following same conditions as for samples. To facilitate visualization, the CypA control data are shown +0.02 µcal, as are the H2AX and dsDNA controls -0.02 µcal. The upper panels show the thermograms for the interactions, whereas the lower panels show the corresponding binding isotherms with integrated heats. Data were fitted to a home-derived model

for a single binding site (continuous lines in binding isotherms). **(F)** Dependence of the measured binding enthalpy (ΔH) for the formation of the $AIF_{\Delta 101}$:dsDNA complex on buffer ionization enthalpy (ΔH_{ion}) at 15 °C. Used buffers were potassium phosphate, TES, MOPS and HEPES at 150 mM ionic strength. The pH was 7.4 and 7.5 respectively in phosphate and TES buffers, and 8.0 for HEPES and MOPS. The solid line represents the fit to a linear regression and provides an estimation of the buffer independent enthalpy (ΔH°) and of the net number of exchanged protons (n_{H+}) upon complex formation. Values show mean ± SD, n=3.

Table S4. Thermodynamic parameters for the formation of binary, ternary and degradosome assemblies.

Sample in the calorimetric cell	Titrating ligand	K_d (μM)	N	ΔH (kcal/mol)	ΔG (kcal/mol)	$-T\Delta S$ (kcal/mol)
			Binary complexes			
**$AIF_{\Delta101}$**	CypA	0.8	0.6	-13.2	-8.3	4.9
**$AIF_{\Delta101}$**	H2AX	0.7	0.6	1.5	-8.1	-9.6
**$AIF_{\Delta101}$**	dsDNA	1.6	0.4	4.9	-7.6	-12.4
**CypA**	dsDNA	8.9	0.6	8.4	-6.7	-15.1
**H2AX**	dsDNA	1.2	0.8	0.4	-7.8	-8.2
			Ternary complexes			
**$AIF_{\Delta101}$:dsDNA (1:1)**	CypA	1.2	0.8	-5.5	-8.1	-2.6
**$AIF_{\Delta101}$:dsDNA (1:2)**	CypA	1.9	0.7	-4.8	-7.8	-3.0
**$AIF_{\Delta101}$:dsDNA (1:4)**	CypA	2.2	0.5	-3.3	-7.7	-4.4
**$AIF_{\Delta101}$:dsDNA (1:8)**	CypA	2.6	0.7	-2.0	-7.6	-5.6
**$AIF_{\Delta101}$:dsDNA (1:1)**	H2AX	0.5	0.7	6.3	-8.3	-14.6
**$AIF_{\Delta101}$:dsDNA (1:2)**	H2AX	0.4	0.7	1.5	-8.5	-10.0
**$AIF_{\Delta101}$:dsDNA (1:4)**	H2AX	0.3	0.7	1.2	-8.6	-10.0
**$AIF_{\Delta101}$:dsDNA (1:8)**	H2AX	0.3	0.7	1.0	-8.7	-9.6
**$AIF_{\Delta101}$:CypA (1:1)**	dsDNA	9.4	0.8	17.2	-6.6	-23.8
**$AIF_{\Delta101}$:CypA (1:8)**	dsDNA	54	1.7	15.6	-5.6	-21.2
**$AIF_{\Delta101}$:H2AX (1:1)**	dsDNA	7.4	0.9	28.9	-6.8	-35.6
**$AIF_{\Delta101}$:H2AX (1:8)**	dsDNA	1.9	1.6	4.2	-7.5	-11.7

			Degradosome			
**AIF$_{\Delta 101}$:CypA**	H2AX	0.8	0.7	1.1	-8.4	-9.5
**AIF$_{\Delta 101}$:H2AX (1:1)**	CypA	2.5	1.2	-26.0	-7.6	18.2
**AIF$_{\Delta 101}$:H2AX (1:8)**	CypA	0.3	1.7	-23.3	-9.0	14.3
			dsDNA-Degradosome			
**AIF$_{\Delta 101}$:CypA:H2AX (1:1:1)**	dsDNA	3.3	0.9	9.9	-7.2	-17.0
**AIF$_{\Delta 101}$:CypA:H2AX (1:1:2)**	dsDNA	1.3	0.9	9.7	-7.8	-17.5
**AIF$_{\Delta 101}$:CypA:H2AX (1:1:4)**	dsDNA	1.0	0.8	9.7	-7.9	-17.6
**AIF$_{\Delta 101}$:CypA:H2AX (1:1:8)**	dsDNA	0.9	1.0	11.9	-8.0	-19.9
**H2AX:dsDNA (1:1)**	AIF$_{\Delta 101}$:CypA	3.6	1.1	-34.1	-7.2	27.0
**H2AX:dsDNA (1:8)**	AIF$_{\Delta 101}$:CypA	3.3	0.8	-39.5	-7.2	32.3

Values obtained from ITC assays at 15 ºC, or at 25 ºC when evaluating interactions in the presence of CypA, in 50 mM potassium phosphate, pH 7.4. N is the calculated binding stoichiometry, usually interpreted as a fraction of binding-competent or active protein. The thermodynamic parameters were calculated using well-known relationships: $K_d = (K_a)^{-1}$, $\Delta G = RT.\ln K_d$ and $-T\Delta S = \Delta G - \Delta H$. Errors considered in the measured parameters (± 30% in K_d and ± 0.4 kcal/mol in ΔH and $-T\Delta S$) were taken larger than the standard deviation between replicates and the numerical error after the fitting analysis.

Binding of dsDNA to each protein was also separately evaluated (**Figure S5C-E**). dsDNA binding was entropically driven with an unfavorable enthalpic contribution in all assayed binary complexes. The interaction was characterized in all cases by a moderate affinity (**Table S4**). Nonetheless, the interactions of AIF$_{\Delta 101}$ and H2AX with dsDNA were stronger in comparison with the CypA:dsDNA one (the later shows a considerable increase in K_d, **Table S4**). In the case of H2AX, this is in particular due to a considerably less unfavorable enthalpic contribution to the binding, despite a just milder favorable entropic contribution. Moreover, these data are consistent with the proposed mechanism of interaction between dsDNA and AIF, which is expected to take place within the clusters of positive charges present throughout the protein's surface (1, 28).

Additional assays were performed with the AIF$_{\Delta 101}$:dsDNA complex in order to estimate the buffer-independent binding enthalpy and the net number of protons

exchanged upon complex formation. Buffers with different ionization enthalpies were employed (**Table S1**), resulting in a buffer-independent ionization free enthalpy of 2.2 cal/mol (**Table S5, Figure S5F**). Additionally, the interaction was observed to be strongly associated with proton exchange between the complex and the buffer, confirming that the affinity is pH–dependent and that about one proton is released into the bulk solution upon complex formation (at least one ionizable group is involved).

Table S5. Thermodynamic parameters determined for the interactions of AIF$_{\Delta 101}$ with dsDNA in different buffers.

**Buffer**	K_d (µM)	**N**	ΔH (kcal/mol)	ΔG (kcal/mol)	$-T\Delta S$ (kcal/mol)
**Phosphate pH 7.4**	1.6	0.4	4.9	-7.6	-12.4
**HEPES pH 8.0**	1.1	0.4	8.5	-7.8	-16.3
**MOPS pH 8.0**	1.0	1.1	8.8	-7.9	-16.7
**TES pH 7.6**	0.1	0.7	9.3	-9.1	-18.4

Values obtained from ITC assays at 15 ºC and at 150 mM ionic strength of the above-mentioned buffers. N is the calculated binding stoichiometry, usually interpreted as a fraction of binding-competent or active protein. The thermodynamic parameters were calculated using well-known relationships: $K_d = (K_a)^{-1}$, $\Delta G = RT \cdot \ln K_d$ and $-T\Delta S = \Delta G - \Delta H$. Errors considered in the measured parameters (± 30% in K_a and ± 0.4 kcal/mol in ΔH and $-T\Delta S$) were taken larger than the standard deviation between replicates and the numerical error after the fitting analysis.

2.2 Ternary and quaternary interactions among the components of the DNA-degradosome

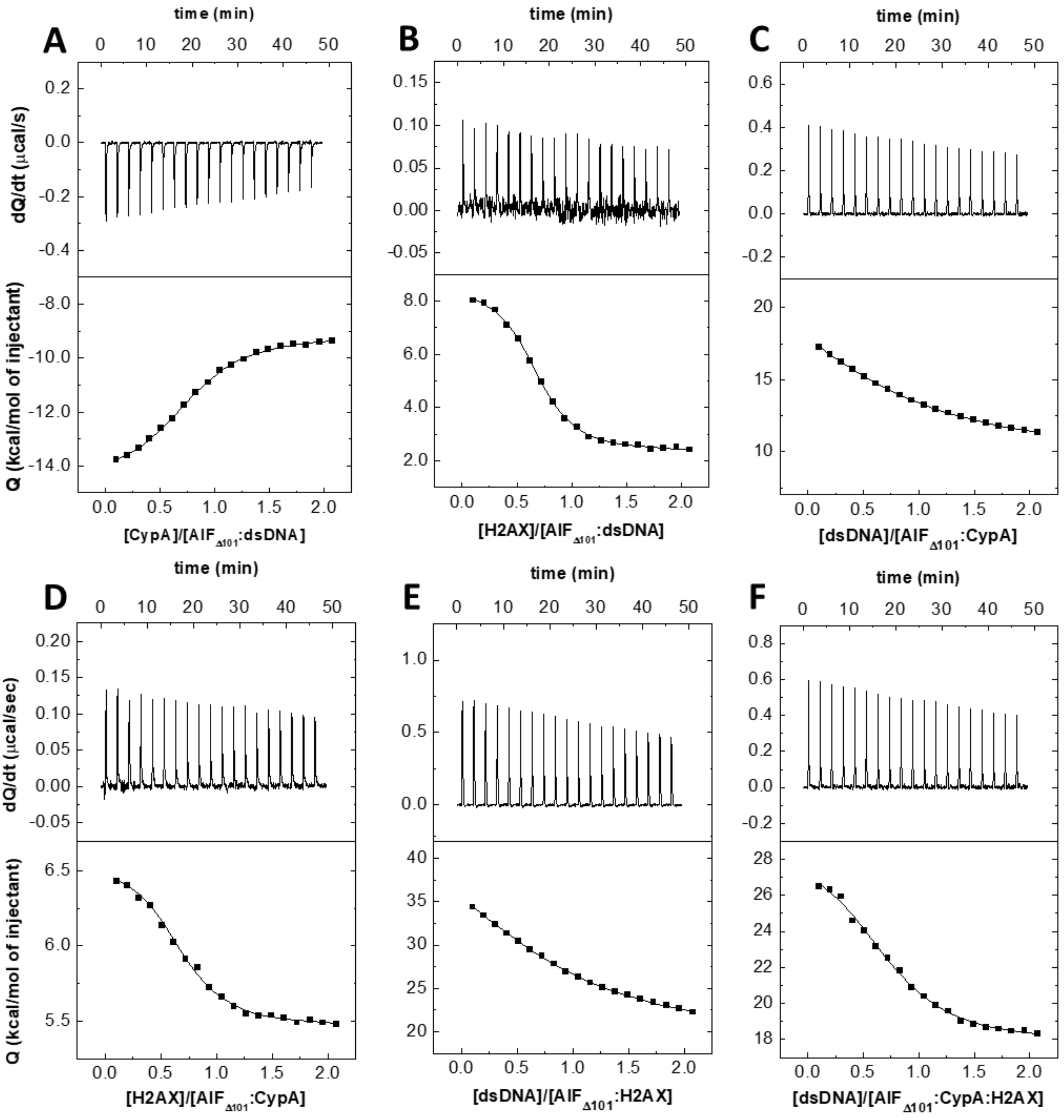

Figure S6. Isothermal calorimetric titrations for ternary and quaternary interactions among the degradosome components. Titration with **(A)** CypA and **(B)** H2AX of AIF$_{\Delta101}$:dsDNA mixtures. Titration with **(C)** dsDNA and **(D)** H2AX of AIF$_{\Delta101}$:CypA mixtures. Titration with (**E**) dsDNA of AIF$_{\Delta101}$:H2AX mixtures. Titration with **(F)** dsDNA of the degradosome. The pre-formed mixtures (1:1÷ ratio) were incubated 15 min in 50 mM potassium phosphate, pH 7.4, at 25 °C. Subsequent ITC measurements were performed in the same buffer at 15 °C, or at 25 ºC when evaluating interactions involving CypA. The upper panels show the

thermograms for the interactions and the lower panels show the corresponding binding isotherms with integrated heats. Data were obtained by fitting ITC thermograms to a home-derived model for a single binding site (continuous lines in binding isotherms)**.**

2.2.1 Electrophoretic-mobility-shift assays and cooperativity effects

Electrophoretic-mobility-shift assays were performed to further evaluate potential differing mechanisms in the formation of the DNA-degradosome (**Figure S7**).

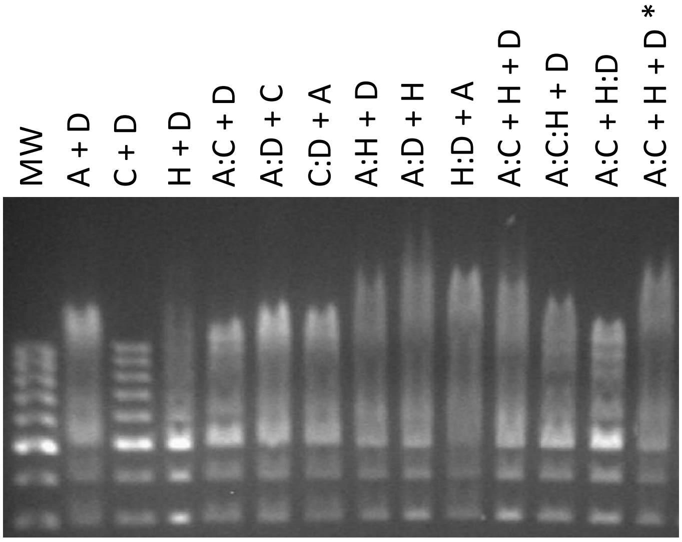

Figure S7. Electrophoretic-mobility-shift assays of the degradosome assemblies. 100 bp DNA ladder (Thermo Scientific) (500 ng) was incubated with individual protein components (10 µg of $AIF_{\Delta101}$, 2 µg of CypA and/or H2AX; as seen in lanes A+D, C+D and H+D respectively) or their combined mixtures in 50 mM potassium phosphate, pH 7.4, at 25 ºC, for 30 minutes, to assess DNA retention. A, C, H and D stand for $AIF_{\Delta101}$, CypA, H2AX and DNA respectively. MW, stands for DNA molecular weight marker. Starting mixtures of two or three components (A:C, A:D, C:D, A:H, A:D, H:D; A:C:H, A:C) were pre-incubated for 30 minutes before mixing with other components (D, C, A, D, H, A, D, pre-incubated H:D, respectively) and incubated again for 30 minutes. Similarly, A:C+H+D, stands for $AIF_{\Delta101}$ and CypA being pre-incubated together for 30 minutes, subsequently mixed with individual samples of H2AX and DNA, and incubated again for additional 30 minutes. A:C+H+D*, stands for $AIF_{\Delta101}$ and CypA being pre-incubated together, then mixed with H2AX and incubated again for 30 minutes, and subsequently mixed with DNA and incubated for another additional 30 minutes. Mixtures were separated by electrophoresis in 2 % agarose gel and visualized with ethidium bromide.

Binary, ternary and quaternary complexes were obtained through the incubation of their components upon their mixing following different sequential additions. Remarkably, incubation of CypA with dsDNA led to no observable dsDNA retention, which only appeared after $AIF_{\Delta101}$ addition. All assessed combinations of CypA and $AIF_{\Delta101}$ resulted in similar modest mobility dsDNA shift effects, with none achieving the one caused by $AIF_{\Delta101}$ alone. This agrees with the observed negative cooperativity for CypA binding to $AIF_{\Delta101}$ in the presence of dsDNA. In

comparison, H2AX exhibited certain dsDNA retention on its own, which underwent a considerable increase in the presence of $AIF_{\Delta 101}$ and is backed by their positive cooperativity. All combinations of $AIF_{\Delta 101}$, H2AX and dsDNA resulted in comparable mobility shift effects. In regard to the evaluated quaternary complexes, the sequential addition of H2AX to a preformed $AIF_{\Delta 101}$:CypA complex and, subsequently, of dsDNA to the resulting $AIF_{\Delta 101}$:CypA:H2AX complex led to the greatest dsDNA retention. Once more, this is supported by the significant positive cooperativity effect observed among the three protein partners, contributing to further confirm the most favorable degradosome assembly order proposed from the ITC data.

2.2.2 Dissecting the DNA-degradosome assembly by AFM

AFM images of mixtures of each isolated protein with dsDNA showed that all proteins remained mainly monomeric (**Table S6**), while topography profiles suggestive of dsDNA interacting with the proteins were observed. Heights corresponding to the protein monomeric features bound to dsDNA, ~9 nm (**Figure S8A**), agreed well with AIF$_{\Delta101}$ (6.3 ± 0.9 nm; **Figure S1A**) plus free dsDNA (1.5 ± 0.5 nm) (**Figure S8J**). Moreover, AIF$_{\Delta101}$ induced the stretching and opening of dsDNA strands (**Figure S8A**). Nonetheless, the binding did not appear to be sequence-specific because distinct sequences along the strands bound AIF$_{\Delta101}$ with similar efficacy. The AIF$_{\Delta101}$:dsDNA interaction also appeared to display cooperativity, since several AIF$_{\Delta101}$ molecules were attached to a DNA strand in a clustered fashion, like “beads on a necklace”, while no condensation of isolated dsDNA strands was observed. **Figure S8B** and **C** also confirmed binding of dsDNA to both CypA and H2AX monomers, according to the height profiles perpendicular to dsDNA molecules (**Table S6**).

When the AIF$_{\Delta101}$:CypA and AIF$_{\Delta101}$:H2AX systems were assayed in the presence of dsDNA, percentages of protein-protein association modes remained in similar ranges as when dsDNA was absent (**Table S6**). Moreover, the morphology and angle of these hetero-dimers were maintained, showing only a few ratios with a smaller angle. Binding of dsDNA to the degradosome increased the percentage of hetero-trimers by nearly two-fold (from 32 up to 52 %), with a modest decrease of hetero-dimers and monomers (32 % and 16 %, respectively; **Table S6**). Hardly any hetero-trimer was found attached to the mica instead of to the dsDNA, as observed by general color scales and verified by height profiles. This observation can be explained by the net charge exhibited by the proteins forming the degradosome, due to differences in terms of their isoelectric points. At working pH, the AIF$_{\Delta101}$ electrical charge must be minimal because its isoelectric point is neutral (29), while CypA is slightly negatively charged (pI 6.4-6.5) and H2AX is positively charged (pI 10.7). Such differences reflect different surface electrostatic potentials inducing protein recognition in specific orientations to enable the hetero-trimer formation. **Figures S8E and 2E** show the hetero-trimer bound to dsDNA in detail.

Table S6. Effect of dsDNA binding on the distribution of quaternary organizations as visualized by AFM imaging of AIF$_{\Delta101}$, CypA and H2AX, as well as of their binary and ternary complexes.

Sample mixtures	Monomers (%)	Hetero-Dimers (%)[a]	Hetero-Trimers (%)	Observed features
**dsDNA**	-	-	-	-
**AIF$_{\Delta101}$:dsDNA**	98	2	-	DNA binding to protein monomers. Stretching and opening of DNA strands
**CypA:dsDNA**	95	5	-	DNA binding to protein monomers
**H2AX:dsDNA**	97	3	-	DNA binding to protein monomers
**AIF$_{\Delta101}$:CypA:dsDNA**	45	55	-	DNA binding to protein hetero-dimers. Stretching of DNA
**AIF$_{\Delta101}$:CypA:dsDNA + Ca^{2+}/Mg^{2+}**	53	47	-	DNA binding to protein hetero-dimers<br>DNA Fragmentation
**AIF$_{\Delta101}$:H2AX:dsDNA**	60	40	--	DNA binding to protein hetero-dimers DNA stretching
**AIF$_{\Delta101}$:CypA:H2AX:dsDNA**	16	32	52	DNA binding to protein complexes
**AIF$_{\Delta101}$:CypA:H2AX:dsDNA + Ca^{2+}/Mg^{2+}**	28	35	37	DNA binding to protein complexes<br>DNA fragmentation

Assays performed in PBS pH 7.0. Percentages are referred to the total number of protein molecules. Concentrations for components in the incubation mixtures were 0.5 µM, 5 mM and 0.05 ng/µl for proteins, divalent cations and dsDNA, respectively. Error is within 5-10 % of the percentage value. [a]Values correspond to homo-dimers in samples containing a single protein.

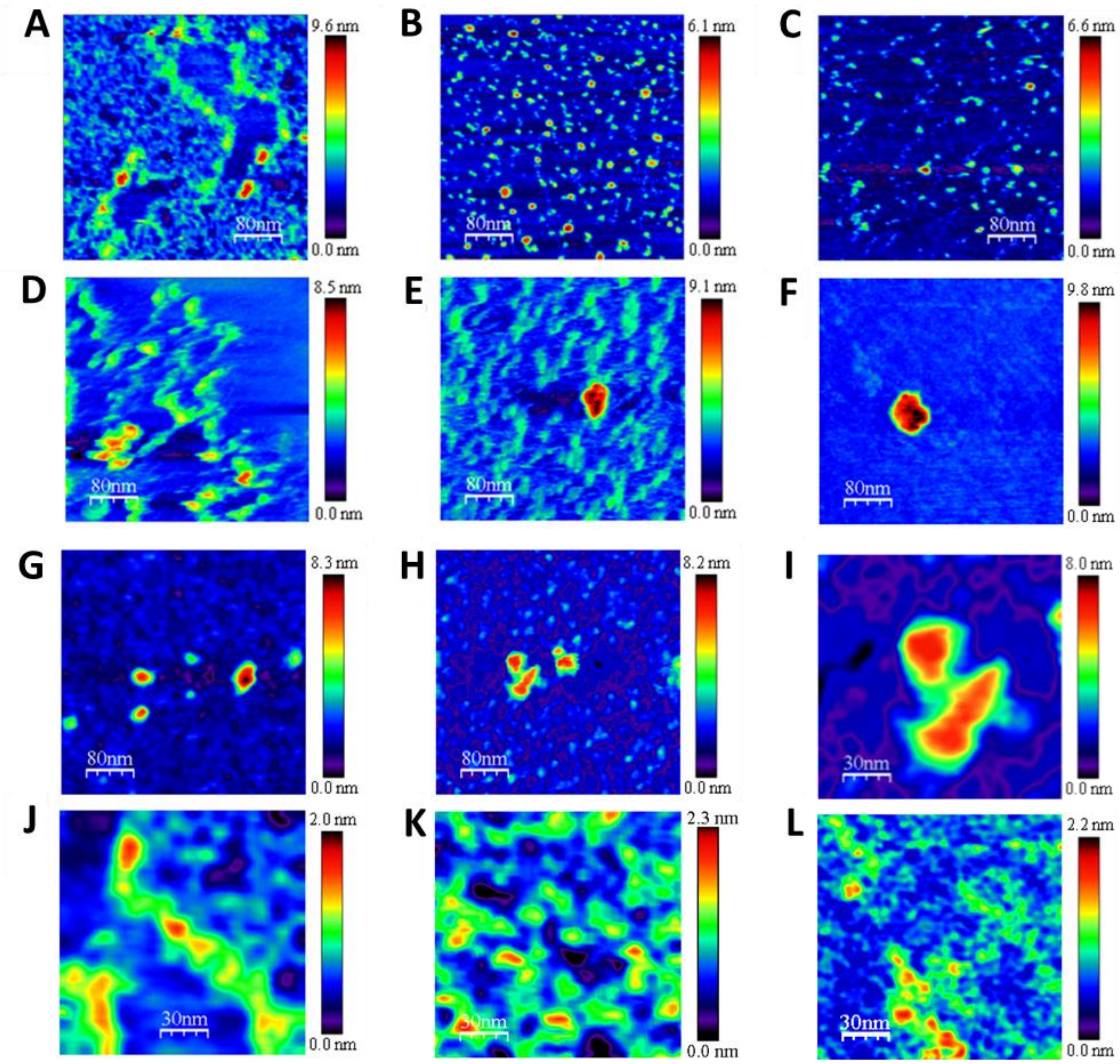

Figure S8. AFM topography of assemblies upon formation of the DNA-degradosome complex. Imaging of samples containing **(A)** $AIF_{\Delta 101}$:dsDNA, **(B)** CypA:dsDNA, **(C)** H2AX:dsDNA, **(D)** $AIF_{\Delta 101}$:CypA:dsDNA, **(E)** $AIF_{\Delta 101}$:H2AX:dsDNA and **(F)** $AIF_{\Delta 101}$:CypA:H2AX:dsDNA assemblies. Impact of Ca^{2+} and Mg^{2+} ions on DNA degradation for **(G)** $AIF_{\Delta 101}$:CypA:dsDNA and **(H)** $AIF_{\Delta 101}$:CypA:H2AX:dsDNA assemblies. **(I)** Zoom image of (H) to evaluate the integrity of the hetero-trimers. **(J)** Linearized dsDNA. (**K**) DNA fragmented by the nuclease activities of $AIF_{\Delta 101}$ and CypA in presence of Ca^{2+} and Mg^{2+} ions. (**L**) dsDNA in $AIF_{\Delta 101}$:CypA:H2AX samples. Scan size 400 nm x 400 nm (A-H) and 150 x 150 nm (I-L). Mixtures of proteins forming the degradosome were prepared as in figure 2E in the presence of 0.05 ng/µl pET-28a(+) plasmid linearized with *Eco*RI.

3. Sources of nuclease activity within the degradosome

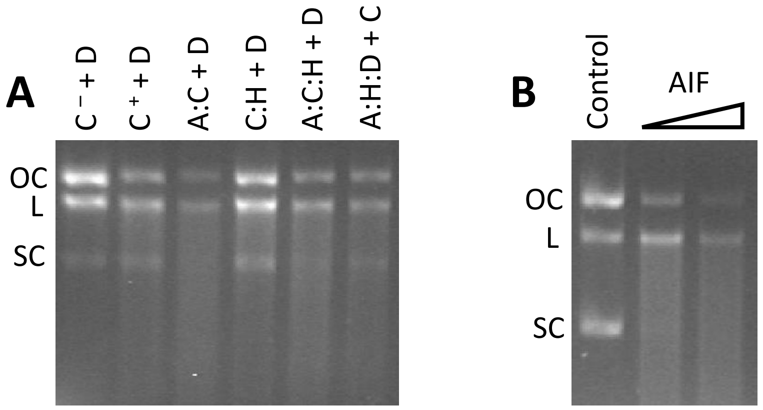

Figure S9. Nuclease activity of the degradosome. (A) Nuclease activity observed upon mixing CypA, individually or in combined mixtures with the degradosome components (250 ng of CypA and/or H2AX; and/or 800 ng of AIF, to maintain 1:1 molar ratios), with double-stranded supercoiled pET-28a(+) plasmid (250 ng). The assays were carried out in 20 mM Tris/HCl, pH 8.0, 1 mM $CaCl_2$ and 1mM $MgCl_2$, and final sample combinations were incubated for 1 hour at 37 ºC. A, C, H and D stand for $AIF_{\Delta 101}$, CypA, H2AX, and plasmidic DNA respectively. C^- and C^+, stand respectively for CypA sample in the absence and presence of ions. Starting mixtures with two or three components (A:C, C:H, A:C:H, A:H:D) were pre-incubated for 15 min at 25 ºC, before mixing with D or C. **(B)** Nuclease activity of $AIF_{\Delta 101}$ (800 or 1600 ng) observed after mixing with double-stranded supercoiled pET-28a(+) plasmid (250 ng) in 20 mM Tris/HCl, pH 8.0, 1 mM $CaCl_2$ and 1mM $MgCl_2$ upon incubating for 1 hour at 37 ºC. Control, plasmid DNA substrate. OC, L and SC, stand for open circular, linear and supercoiled.

3.1 AIF preparations have no adventitious nuclease contamination

Purity of $AIF_{\Delta 101}$ protein samples was confirmed by SDS-PAGE (**Figure S10A**) and mass spectrometry (MS) assays. Most peptides identified by MS correspond to human AIF, and no nuclease peptide from any other sources was identified. Additionally, the purity of the AIF protein samples was confirmed through CN-PAGE, proving that there was no nuclease contamination. To do so, a purified $AIF_{\Delta 101}$ sample was separated by high-resolution CN-PAGE in duplicate lanes. One lane was subsequently stained with Coomassie blue (**Figure S10B**) and the corresponding band of $AIF_{\Delta 101}$ was excised from the duplicate lane. The excised band was then incubated with 250 ng of plasmid DNA for 5 min at 37 ºC. Two additional portions of the same lane above and below the $AIF_{\Delta 101}$ band - corresponding to higher and lower molecular weights- were also assayed, as was an additional portion of an empty lane from the same gel. The latter demonstrated a destabilizing effect on dsDNA upon incubation with polyacrylamide, resulting in the apparition of the linear and open circular forms that could be observed in all samples (**Figure S10B**). However, no smearing was apparent in any sample excepting that of the $AIF_{\Delta 101}$ band, confirming that the source of the observed nuclease activity is indeed AIF.

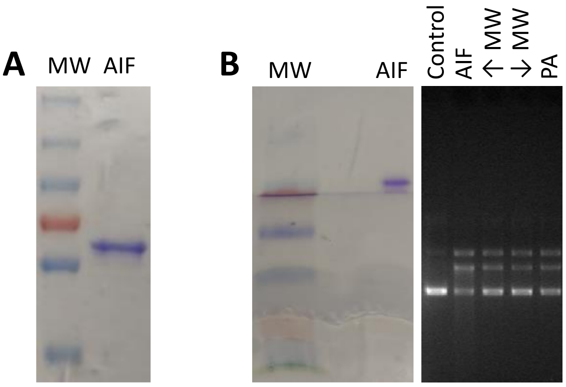

Figure S10. AIF preparations have no adventitious nuclease contamination. **(A)** SDS-PAGE of $AIF_{\Delta 101}$ after purification to homogeneity. **(B)** CN-PAGE pattern of $AIF_{\Delta 101}$ (AIF, 250 ng) after Coomassie blue staining (left panel) and nuclease activity of the corresponding excised band of the $AIF_{\Delta 101}$ protein from a CN-PAGE duplicate lane upon mixing with double-stranded supercoiled pET-28a(+) plasmid (250 ng) in 20 mM Tris/HCl, pH 8.0, 0.1 mM $CaCl_2$ and 1mM $MgCl_2$ for 5 minutes at 37ºC (right panel). Portions of gel above or below the AIF band corresponding to higher or lower MWs, and labelled as lanes ↑ MW and ↓ MW respectively, were similarly incubated with pET-28a(+) and used as negative controls. Additionally, a portion of polyacrylamide gel from a lane without loaded AIF sample was incubated with pET-28a(+) (PA lane). MW, protein molecular weight marker.

3.2 Human and mouse AIF share nuclease activity

The detected AIF nuclease activity was also confirmed with purified mouse AIF (mAIF) using both plasmid and genomic DNA substrates under similar conditions to those for the human protein (**Figure S11**). These assays confirmed that mAIF also presents a certain degree of nuclease activity, although being significantly less efficient than human AIF. An assessment of different enhancing divalent ions (Ca^{2+}, Mg^{2+} and Mn^{2+}) demonstrated that the nuclease activity of mAIF becomes optimal with 0.1 mM of Mn^{2+} (**Figure S11A**), differing again from human AIF.

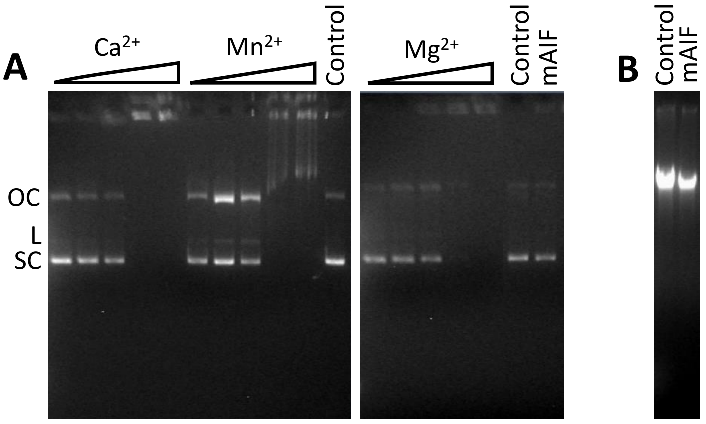

Figure S11. Nuclease activity of mouse AIF. (A) Effect of different ions expected to promote (Ca^{2+}, Mg^{2+} and Mn^{2+}) or inhibit (K^+ and Na^+) the *in vitro* nuclease activity of mAIF (250 ng). Increasing ion concentrations, 0.01, 0.1, 1, 10 or 100 mM, were evaluated while incubating AIF with double-stranded supercoiled pET-28a(+) plasmid (250 ng) in 20 mM Tris/HCl, pH 8.0, for 1 hour at 37 ºC. OC, L and SC, stand for open circular, linear and supercoiled. **(B)** Nuclease activity of mAIF (250 ng) assessed against mouse genomic DNA (500 ng) after incubating for 4 h at 37 ºC in the presence of 0.1 mM $MnCl_2$. Control stands for either plasmid or genomic DNA substrate. mAIF stands for either plasmid or genomic DNA substrate incubated with mAIF in absence of ions (panel A) or with 0.1 mM $MnCl_2$ (panel B).

3.3 The influence of partners and key residues on AIF nuclease activity

The protein partners' influence on $AIF_{\Delta101}$ nuclease activity was further investigated using genomic DNA ScreenTape (*Agilent*) to determine the size of the remaining DNA, the concentration of intact dsDNA and the DNA Integrity Number (DIN) (**Table S7**). These data are fully discussed in the main text.

Table S7. Nuclease activity of $AIF_{\Delta101}$ and its TopIB and DEK variants on Genomic DNA.

Sample	Peak (kbp)	[55- >60 kbp]* (%)	DIN
**dsDNA**	>60	82	9.5
**$AIF_{\Delta101}$ variant**			
**WT**	26	9	7.7
**CypA**	51	21	8.1
**H2AX**	>60	52	8.9
**WT:CypA**	21	7	7.0
**WT:H2AX**	56	30	8.4
**WT:CypA:H2AX**	21	4	6.9
**WT:CypA + H2AX:dsDNA**	25	11	7.2
**WT:CypA + H2AX + dsDNA**	23	7	7.1
**WT + CypA + H2AX:dsDNA**	18	8	6.6
**Y443A**	>60	60	9.6
**K446A**	>60	60	9.6
**R449A**	>60	64	9.7
**R450A**	>60	64	9.5
**R451A**	58	39	8.7
**H454S**	>60	41	8.7
**D489A/K518A**	>60	45	8.4
**K518A/E522A**	>60	42	8.3
**K510A/K518A**	42	17	6.9

Genomic DNA integrity after nuclease activity assays was analyzed using the Genomic DNA ScreenTape assay on the 2200 TapeStation microfluidic platform (Genomic ScreenTape device, Agilent Technologies). Samples were prepared as in the solution nuclease assays (250 ng of protein and 500 ng of genomic DNA), but were incubated for 5 min at room temperature. DIN: DNA Integrity Number.

3.4 Effects of the variants of key residues for AIF's nuclease activity

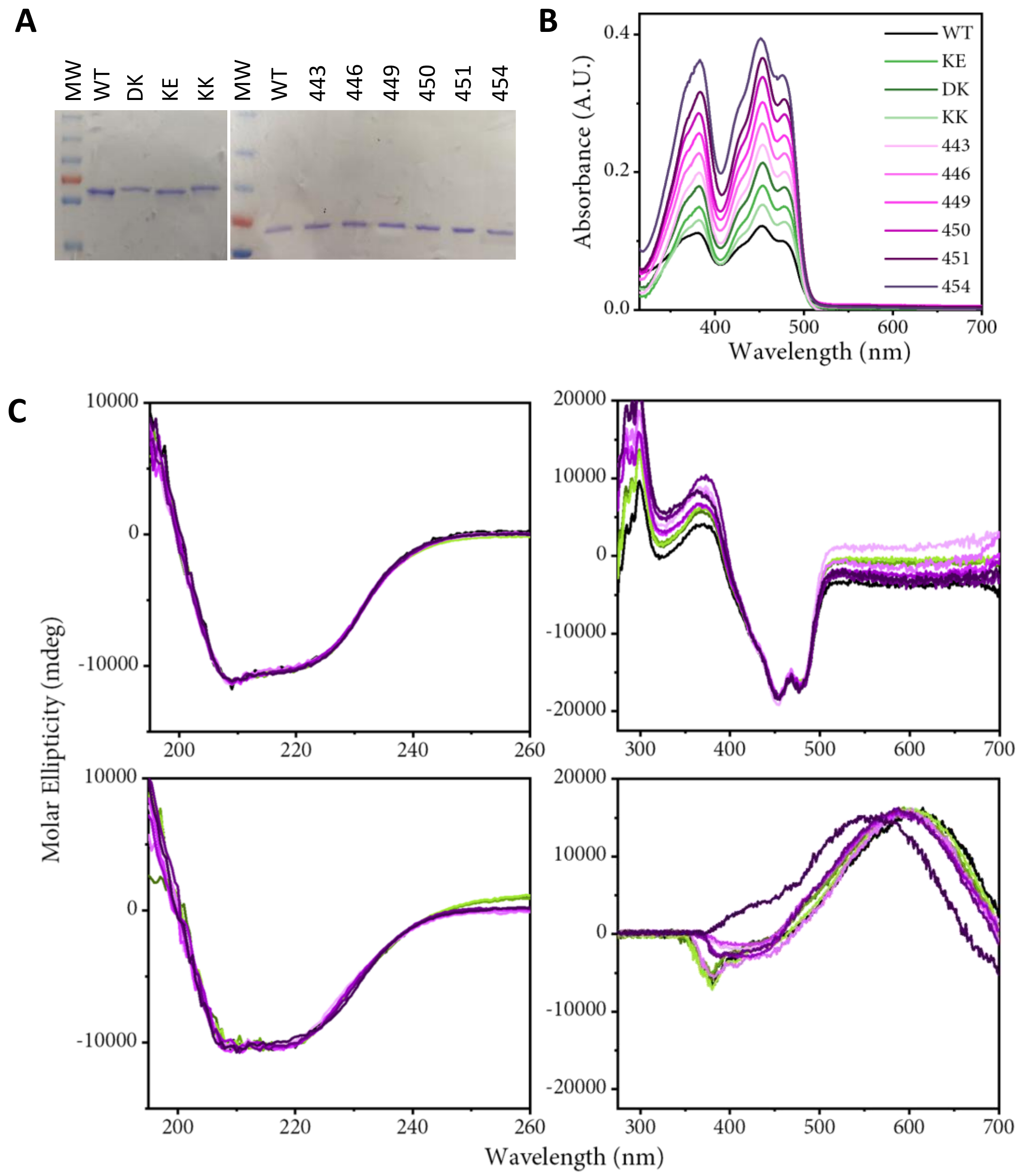

Figure S12. SDS-PAGE, visible absorption and circular dichroism spectra of the TopIB and DEK variants of AIF. (A) SDS-PAGE of AIF$_{\Delta 101}$ WT and variants after purification to homogeneity. **(B)** Visible absorption spectra of purified AIF$_{\Delta 101}$ WT and variants recorded in 50 mM potassium phosphate, pH 7.4, at 25 °C. Different protein concentrations were employed to facilitate visualization. **(C)** CD spectra of AIF$_{\Delta 101}$ WT and variants in the far–UV (2 µM samples) and in the near–UV/Vis (20 µM samples) are shown in left and right panels respectively. The assays were performed in the absence (upper) and presence of 100–fold excess NADH (lower). WT AIF$_{\Delta 101}$ is shown in black, variants D489A/K518A, K518A/E522A and K510A/K518A in increasingly darker shades of green, and variants Y443A, K446A, R449A, R450A, R451A and H454S in increasingly darker shades of purple. Code for mutated residue in AIF$_{\Delta 101}$ variants: 443, Y443A; 446, K446A; 449, R449A; 450, R450A; 451, R451A; 454, H454S; DK, D489A/K518A; KE, K518A/E522A; KK, K510A/K518A.

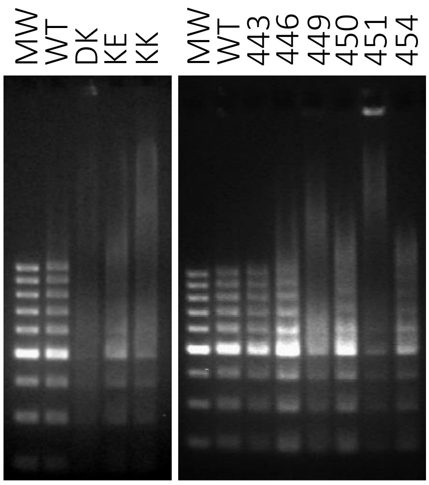

Figure S13. Effect of TopIB and DEK mutations on DNA retention by AIF. 100 bp DNA ladder (500 ng), MW, (Thermo Scientific) were incubated with AIF$_{\Delta 101}$ WT or its variants (6 µg) for 30 min in 50 mM potassium phosphate, pH 7.4, at 25 ºC, to assess DNA retention. Code for mutated residues in AIF$_{\Delta 101}$ variants: 443, Y443A; 446, K446A; 449, R449A; 450, R450A; 451, R451A; 454, H454S; DK, D489A/K518A; KE, K518A/E522A; KK, K510A/K518A. Mixtures were separated by electrophoresis in 2 % agarose gel and visualized with ethidium bromide.

Table S8. Thermodynamic parameters for the binary interaction of $AIF_{\Delta101}$ variants with dsDNA.

$AIF_{\Delta101}$ variant	Titrating ligand	K_d (μM)	ΔH (kcal/mol)	ΔG (kcal/mol)	$-T\Delta S$ (kcal/mol)
**WT**	dsDNA	2.9	5.9	-7.3	-13.2
**Y443A**	dsDNA	3.0	8.4	-7.2	-15.6
**K446A**	dsDNA	3.0	5.7	-7.3	-13.0
**R449A**	dsDNA	2.3	5.2	-7.4	-12.6
**R450A**	dsDNA	2.9	7.4	-7.3	-14.7
**R451A**	dsDNA	2.0	4.7	-7.5	-12.2
**H454A**	dsDNA	2.4	6.3	-7.4	-13.6
**K510A/K518A**	dsDNA	0.5	3.8	-8.3	-12.1
**D489A/K518A**	dsDNA	0.5	2.5	-8.3	-10.8
**K518A/E522A**	dsDNA	5.7	10.6	-6.9	-17.5

Values obtained from ITC assays at 15 ºC in 50 mM potassium phosphate, pH 7.4. N is the calculated stoichiometry for binding. The thermodynamic parameters were calculated using well-known relationships: $K_d = (K_a)^{-1}$, $\Delta G = RT.\ln K_d$ and $-T\Delta S = \Delta G - \Delta H$. Errors considered in the measured parameters (± 30% in K_d and ± 0.4 kcal/mol in ΔH and $-T\Delta S$) were taken larger than the standard deviation between replicates and the numerical error after the fitting analysis.

3.5 Insights into the molecular mechanism of AIF nuclease activity

Since mutations at DEK and TopIB motifs do not have a major effect on dsDNA binding, their negative effect on the direct AIF ability to degrade genomic DNA (**Table SP7**) has to relate with these residues being somehow implicated on the nuclease catalytic process. To better illustrate this possibility at the molecular level, relative orientations of residues at the DEK and TopIB motifs regarding dsDNA were evaluated in dsDNA:AIF docking models built by HADDOCK as above described. Top panel in **Figure S14A** shows the potential organization upon binding of dsDNA to the AIF DEK motif through divalent cations. In AIF this motif appears to occur in a β-hairpin plus an α-helix. DEK is a divalent cation dependent-motif that has been shown to adapt to diverse surrounding tertiary structures in different nuclease activities and pathways by being diverse and permissive in primary sequence (30). In this motif, negatively charged residues contribute to fit the positions of divalent cations that bind the target dsDNA. On its side, the third residue, Lys in AIF, usually H-bonds to a nucleophilic water and to the dsDNA, and is attributed to couple the recognition of the target DNA sequence with the cleavage reaction (31). As shown in medium and bottom panels of **Figure S14A**, Ala replacements of any of these three residues will surely alter either the DEK motif nuclease catalytic step or the achievement of the competent geometry for it to occur. Regarding the AIF TopIB active site, the top panel in **Figure S14B** shows that it contains the expected Arg, Lys, His and nucleophile Tyr residues for this motif, with the basic residues oriented to neutralize the DNA backbone (32, 33). The model shows that the highly AIF conserved R449 can H-bond both the scissile DNA phosphate and the postulated nucleophile Y443, while the other charged residues might contribute as general acids to protonate the 5' leaving group of the DNA. Again, as shown in the middle and bottom panels of **Figure S14B**, Ala replacements at any of these residues will break the sequential events proposed for a TopIB motif nuclease activity.

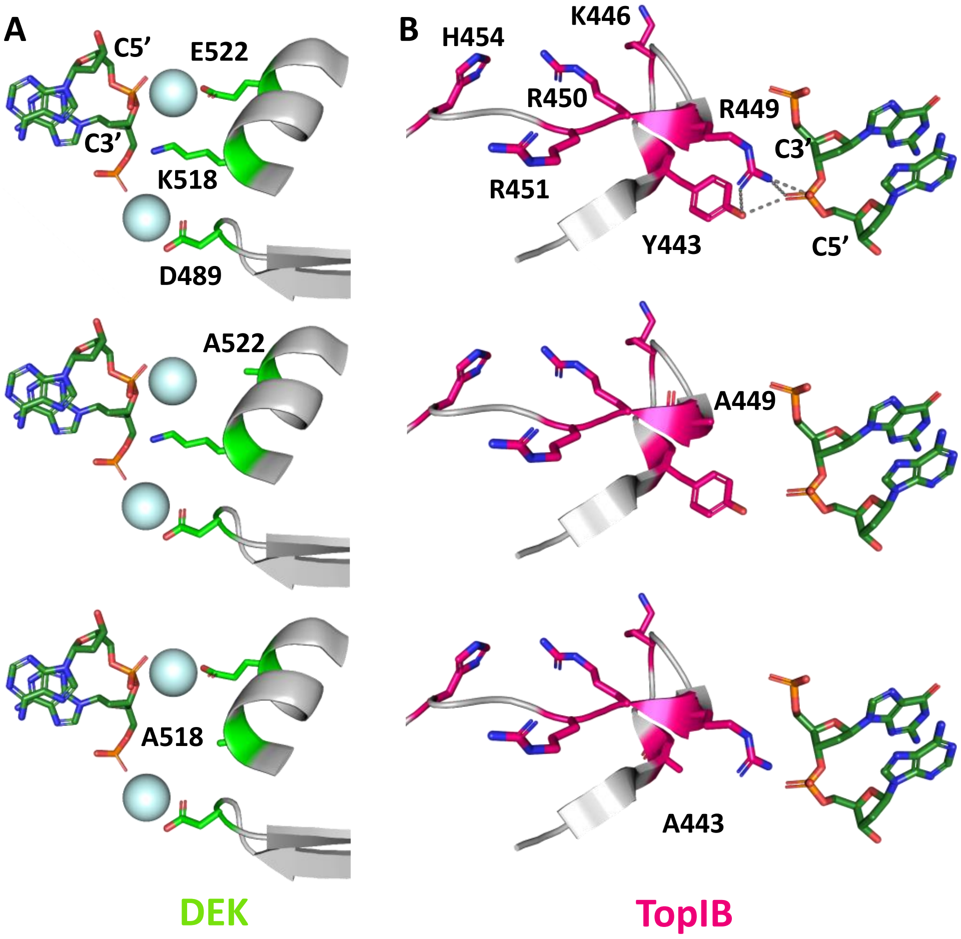

Figure S14. Molecular simulation of the impact of mutations at DEK and TopIB motifs of AIF on its interplay with DNA molecules. (A) Model for a potential organization of the AIF DEK motif and the target DNA during DEK nuclease activity. The top panel shows the WT model, while middle and bottom panels represent the potential impact of the E522A and K518A mutations. Removal of any of the three side-chains of the DEK motif will negatively impact the structural DEK-DNA-divalent cation organization. **(B)** Model for a potential organization of the AIF TopIB motif and the target DNA during TopIB nuclease activity. The top panel shows the WT model, while medium and bottom panels represent potential impact of the R449A and Y443A mutations. Removal of these side-chains will prevent achievement of catalytic competent geometry expected for TopIB nuclease activity. Residues of the DEK and TopIB motifs are highlighted in sticks with carbons respectively colored in green and magenta. Target nucleotides from the docked DNA chain to each motif are in sticks CPK colored with carbons in dark green. The DEK motif shows in pale blue spheres a potential position for the two divalent cations (placed as observed in other DEK motifs to compensate the acidic residues), while in the TopIB the top WT panel highlights as dashed lines the proposed interplay among R449, Y443 and the target DNA phosphate occurring during nuclease activity. dsDNA:AIF models are shown as produced by the HADDOCK 2.4 web server using dsDNA of 15 to 20 bp sequences as ligands and the conformation of AIF in the energetically optimized degradosome model as receptor (**Figures S3 and S4**).

In agreement with the mutated residues at the TopIB and DEK motifs being at the protein surface, Ala substitutions do not alter protein conformation (**Figure S14**). Therefore, mutant models directly produced on the energetically optimized WT AIF molecular model are adequate to evaluate the impact of the mutations on the protein electrostatic surface potential (ESP). As shown in **Figure S15**, replacements to Ala produced very minor changes in the overall protein ESP, with subtle changes being only observed for some of the mutants at the position of the introduced mutation. Considering that the binding of dsDNA to AIF is non-specific and contributed by several residues and regions on the protein surface, this agrees with none of the mutations preventing dsDNA binding and with some even favoring it (**Table S8**).

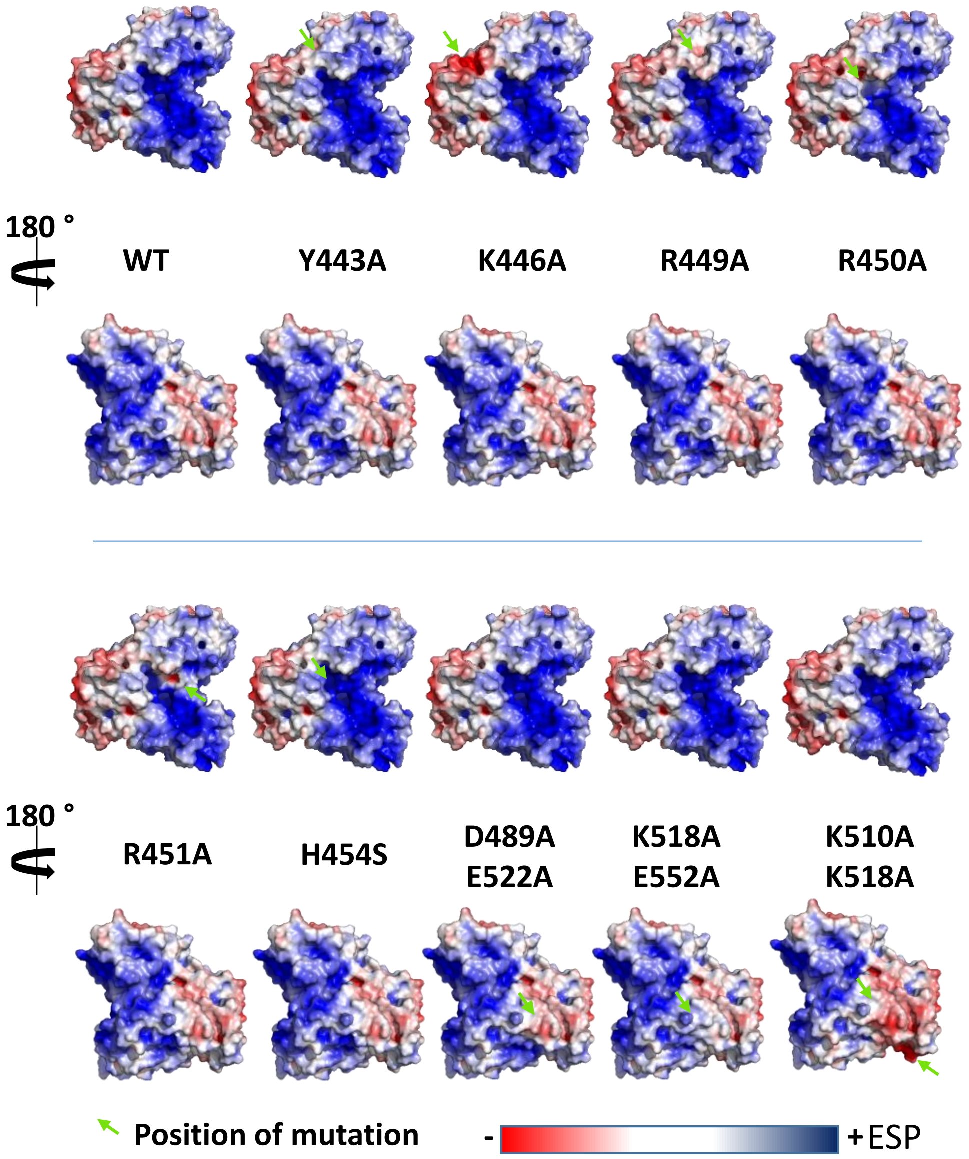

Figure S15. Impact of mutations at the TopIB and DEK motifs on the AIF electrostatic surface potential (ESP). ESP for each variant was calculated at pH 7.0 using the APBS-PDB2PQR software suite (https://www.poissonboltzmann.org/) and then plotted using PyMOL. Position of mutation(s) for each variant is indicated by a green arrow.

1. S. Romero-Tamayo *et al.*, W196 and the *β*-Hairpin Motif Modulate the Redox Switch of Conformation and the Biomolecular Interaction Network of the Apoptosis-Inducing Factor. *Oxid Med Cell Longev* **2021**, 6673661 (2021).
2. P. Ferreira *et al.*, Structural Insights into the Coenzyme Mediated Monomer–Dimer Transition of the Pro-Apoptotic Apoptosis Inducing Factor. *Biochemistry* **53**, 4204-4215 (2014).
3. A. Lostao, M. Medina, Atomic Force Microscopy: Single-Molecule Imaging and Force Spectroscopy in the Study of Flavoproteins Ligand Binding and Reaction Mechanisms. *Methods Mol Biol* **2280**, 157-178 (2021).
4. M. C. Pallarés *et al.*, Sequential binding of FurA from Anabaena sp. PCC 7120 to iron boxes: Exploring regulation at the nanoscale. *Biochimica et Biophysica Acta (BBA) - Proteins and Proteomics* **1844**, 623-631 (2014).
5. A. Lostao, M. L. Peleato, C. Gómez-Moreno, M. F. Fillat, Oligomerization properties of FurA from the cyanobacterium Anabaena sp. PCC 7120: Direct visualization by in situ atomic force microscopy under different redox conditions. *Biochimica et Biophysica Acta (BBA) - Proteins and Proteomics* **1804**, 1723-1729 (2010).
6. I. Horcas *et al.*, WSXM: A software for scanning probe microscopy and a tool for nanotechnology. *Review of Scientific Instruments* **78**, 013705 (2007).
7. N. Vahsen *et al.*, Physical interaction of apoptosis-inducing factor with DNA and RNA. *Oncogene* **25**, 1763-1774 (2006).
8. R. N. Goldberg, N. Kishore, R. M. Lennen, Thermodinamic quantities for the ionization reactions of buffers. *J. Phys. Chem. Ref. Data* **31**, 231-370 (2002).
9. M. Martinez-Julvez, O. Abian, S. Vega, M. Medina, A. Velazquez-Campoy, "Studying the Allosteric Energy Cycle by Isothermal Titration Calorimetry" in Allostery: Methods and Protocols, A. W. Fenton, Ed. (Springer New York, New York, NY, 2012), pp. 53-70.
10. A. Velazquez-Campoy, G. Goñi, J. R. Peregrina, M. Medina, Exact analysis of heterotropic interactions in proteins: Characterization of cooperative ligand binding by isothermal titration calorimetry. *Biophys J* **91**, 1887-1904 (2006).
11. B. Jiménez-García, C. Pons, J. Fernández-Recio, pyDockWEB: a web server for rigid-body protein-protein docking using electrostatics and desolvation scoring. *Bioinformatics* **29**, 1698-1699 (2013).
12. M. J. Abraham *et al.*, Gromacs: High performance molecular simulations through multi-level parallelism from laptops to supercomputers. *SoftwareX* **1-2**, 19-25 (2015).
13. B. Farina *et al.*, Structural and biochemical insights of CypA and AIF interaction. *Sci Rep* **7**, 1138 (2017).
14. M. Baritaud, H. Boujrad, H. K. Lorenzo, S. Krantic, S. A. Susin, Histone H2AX: The missing link in AIF-mediated caspase-independent programmed necrosis. *Cell Cycle* **9**, 3166-3173 (2010).
15. M. H. Olsson, C. R. Søndergaard, M. Rostkowski, J. H. Jensen, PROPKA3: Consistent Treatment of Internal and Surface Residues in Empirical pKa Predictions. *J Chem Theory Comput* **7**, 525-537 (2011).
16. M. J. Frisch *et al.* (2016) Gaussian 09, Revision A.02. (Gaussian, Inc., Wallingford CT).
17. W. Humphrey, A. Dalke, K. Schulten, VMD: visual molecular dynamics. *J Mol Graph* **14**, 33-38, 27-38 (1996).
18. W. L. Delano, The PyMOL molecular graphics system. *DeLano Scientific, San Carlos, CA, USA*, http://www.pymol.org (2002).
19. G. C. P. van Zundert *et al.*, The HADDOCK2.2 Web Server: User-Friendly Integrative Modeling of Biomolecular Complexes. *J Mol Biol* **428**, 720-725 (2016).
20. S. Hornus, B. Lévy, D. Larivière, E. Fourmentin, Easy DNA modeling and more with GraphiteLifeExplorer. *PLoS One* **8**, e53609 (2013).

21. N. Novo, P. Ferreira, M. Medina, The apoptosis-inducing factor family: Moonlighting proteins in the crosstalk between mitochondria and nuclei. *IUBMB Life* **73**, 568-581 (2021).
22. M. Lammers, H. Neumann, J. W. Chin, L. C. James, Acetylation regulates cyclophilin A catalysis, immunosuppression and HIV isomerization. *Nat Chem Biol* **6**, 331-337 (2010).
23. F. Favretto *et al.*, The Molecular Basis of the Interaction of Cyclophilin A with α-Synuclein. *Angew Chem Int Ed Engl* **59**, 5643-5646 (2020).
24. T. Saleh *et al.*, Cyclophilin A promotes cell migration via the Abl-Crk signaling pathway. *Nat Chem Biol* **12**, 117-123 (2016).
25. M. Dujardin, J. Bouckaert, P. Rucktooa, X. Hanoulle, X-ray structure of alisporivir in complex with cyclophilin A at 1.5 Å resolution. *Acta Crystallogr F Struct Biol Commun* **74**, 583-592 (2018).
26. L. Jin, S. C. Harrison, Crystal structure of human calcineurin complexed with cyclosporin A and human cyclophilin. *Proc Natl Acad Sci U S A* **99**, 13522-13526 (2002).
27. I. Salguero *et al.*, MDC1 PST-repeat region promotes histone H2AX-independent chromatin association and DNA damage tolerance. *Nat Commun* **10**, 5191 (2019).
28. H. Ye *et al.*, DNA binding is required for the apoptogenic action of apoptosis inducing factor. *Nat Struct Biol* **9**, 680-684 (2002).
29. C. Candé, F. Cecconi, P. Dessen, G. Kroemer, Apoptosis-inducing factor (AIF): key to the conserved caspase-independent pathways of cell death? *Journal of cell science* **115**, 4727 (2002).
30. W. Yang, Nucleases: diversity of structure, function and mechanism. *Q Rev Biophys* **44**, 1-93 (2011).
31. J. Y. Lee *et al.*, MutH complexed with hemi- and unmethylated DNAs: coupling base recognition and DNA cleavage. *Mol Cell* **20**, 155-166 (2005).
32. L. Yakovleva, S. Chen, S. M. Hecht, S. Shuman, Chemical and traditional mutagenesis of vaccinia DNA topoisomerase provides insights to cleavage site recognition and transesterification chemistry. *J Biol Chem* **283**, 16093-16103 (2008).
33. D. R. Davies, A. Mushtaq, H. Interthal, J. J. Champoux, W. G. Hol, The structure of the transition state of the heterodimeric topoisomerase I of Leishmania donovani as a vanadate complex with nicked DNA. *J Mol Biol* **357**, 1202-1210 (2006).
